# CD8^+^ lymphocytes do not impact SIV reservoir establishment under ART

**DOI:** 10.1038/s41564-022-01311-9

**Published:** 2023-01-23

**Authors:** Maura Statzu, Wang Jin, Emily J. Fray, Andrew Kam Ho Wong, Mithra R. Kumar, Elizabeth Ferrer, Steffen S. Docken, Mykola Pinkevych, Julia B. McBrien, Christine M. Fennessey, Brandon F. Keele, Shan Liang, Justin L. Harper, Simona Mutascio, Lavinia Franchitti, Hong Wang, Davide Cicetti, Steven E. Bosinger, Diane G. Carnathan, Thomas H. Vanderford, David M. Margolis, J. Victor Garcia-Martinez, Ann Chahroudi, Mirko Paiardini, Janet Siliciano, Miles P. Davenport, Deanna A. Kulpa, Robert S. Siliciano, Guido Silvestri

**Affiliations:** 1 Emory National Primate Research Center, Department of Pathology and Laboratory Medicine, and Emory Vaccine Center, Emory University, Atlanta, GA USA; 2 Kirby Institute, University of New South Wales, Sydney, Australia; 3 Department of Medicine, Johns Hopkins University School of Medicine, Baltimore, MD USA; 4 AIDS and Cancer Virus Program, Frederick National Laboratory for Cancer Research, Frederick, MD USA; 5 Division of Infectious Diseases, Center for AIDS Research, University of North Carolina at Chapel Hill, School of Medicine, Chapel Hill, NC USA; 6 Department of Pediatrics, Emory University, Atlanta, GA USA

**Keywords:** HIV infections, Viral reservoirs

## Abstract

Persistence of the human immunodeficiency virus type-1 (HIV-1) latent reservoir in infected individuals remains a problem despite fully suppressive antiretroviral therapy (ART). While reservoir formation begins during acute infection, the mechanisms responsible for its establishment remain unclear. CD8^+^ T cells are important during the initial control of viral replication. Here we examined the effect of CD8^+^ T cells on formation of the latent reservoir in simian immunodeficiency virus (SIV)-infected macaques by performing experimental CD8^+^ depletion either before infection or before early (that is, day 14 post-infection) ART initiation. We found that CD8^+^ depletion resulted in slower decline of viremia, indicating that CD8^+^ lymphocytes reduce the average lifespan of productively infected cells during acute infection and early ART, presumably through SIV-specific cytotoxic T lymphocyte (CTL) activity. However, CD8^+^ depletion did not change the frequency of infected CD4^+^ T cells in the blood or lymph node as measured by the total cell-associated viral DNA or intact provirus DNA assay. In addition, the size of the persistent reservoir remained the same when measuring the kinetics of virus rebound after ART interruption. These data indicate that during early SIV infection, the viral reservoir that persists under ART is established largely independent of CTL control.

## Abstract (web summary)

Depletion of CD8^+^ T cells before infection or early antiretroviral therapy initiation elucidates the role of cytotoxic T lymphocytes during formation of the latent simian immunodeficiency virus reservoir.

## Main

The critical barrier to an HIV cure is a population of latently infected cells harbouring integrated replication-competent virus (that is, ‘viral reservoir’) that persists indefinitely in antiretroviral therapy (ART)-treated human immunodeficiency virus (HIV)-infected people^1–3^. The mechanisms responsible for the establishment and maintenance of the reservoir remain incompletely understood in both HIV infection of humans and simian immunodeficiency virus (SIV) infection of rhesus macaques (*Macaca mulatta*, RMs), which represents the most used non-human primate model for HIV infection and AIDS^4^.

Many lines of evidence indicate that CD8^+^ T-cell-mediated cytotoxic T lymphocyte (CTL) activity plays an important role in controlling virus replication during acute HIV/SIV infection^5^. First, the post-peak decline of viremia occurs after the emergence of virus-specific CD8^+^ T cells, suggesting that these cells are involved in the initial control of viral replication^6,7^. Second, viral mutants capable of escaping the CD8^+^ T-cell response rapidly become fixed in the virus population, thus demonstrating a strong evolutionary pressure imposed on the virus by CD8^+^ T cells^8–11^. Third, depletion of CD8^+^ T cells during acute SIV infection results in the abrogation of the post-peak decline of viremia^12,13^. Of note, recent studies also suggest that CD8^+^ T cells inhibit virus production through non-cytolytic mechanisms that suppress HIV/SIV transcription^14,15^, thus leading to a novel paradigm according to which CD8^+^ T cells control viremia through both ‘canonical’ CTL and non-cytolytic suppression of virus production^5^.

In this study, we used the previously validated in vivo experimental system of antibody-mediated CD8^+^ lymphocyte depletion^16,17^ to study the mechanisms responsible for the establishment of the virus reservoir in SIV-infected, ART-treated RMs. The key result of this study is that CD8^+^ lymphocytes do not impact SIV reservoir establishment under ART.

## Results

### Slower decay of viremia after CD8^+^ lymphocyte depletion

**Fig. 1:**
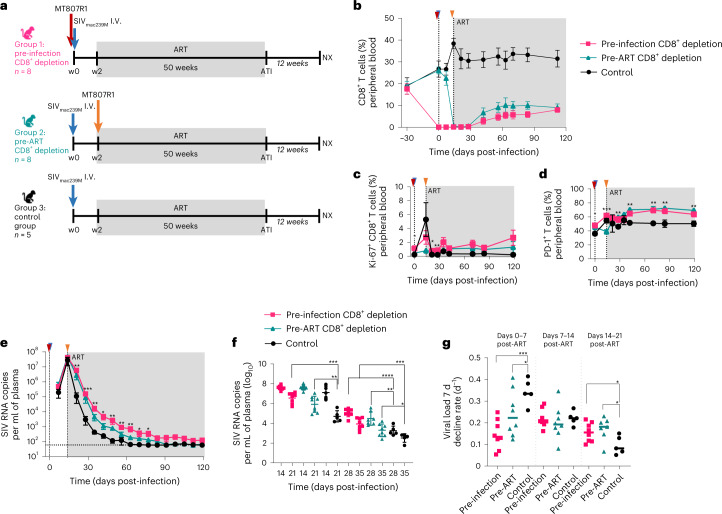
Study design, CD8^+^ T lymphocyte depletion and SIV plasma viral load kinetics. **a**, Study design. **b**, CD8^+^ T-cell kinetics in peripheral blood after depletion. **c**,**d**, Longitudinal flow cytometry analysis of Ki-67 (**c**) or PD-1 (**d**) expression in bulk CD8^+^ T cells after CD8^+^ T-cell depletion in the Pre-infection depletion group (*n* = 8 macaques, magenta line), Pre-ART group (*n* = 8 macaques, teal line) and in the control group (*n* = 5 macaques, black line). **e**, SIV plasma viral load in the first 120 d p.i. in the three experimental groups (*n* = 21 macaques). Limit of detection is 60 copies of SIV RNA per ml of plasma (horizontal dotted line). **f**, Plasma viral load comparison among the 3 groups at days 14 and 21 (left side) and days 28 and 35 (right side). **g**, Viral load 7 d decline rate. In **a**–**e**: blue arrow indicates SIVmac239M infection; red and orange arrows indicate the administration of 50 mg kg^−1^ MT807R1 before infection or ART initiation, respectively. Grey box represents time on ART. In **b**–**e**: data are mean ± s.e.m. Kruskal Wallis test was used to compare the values between the groups. In **b**–**g**: magenta squares/lines represent the Pre-Infection group (*n* = 8 macaques), teal triangles/lines represent the Pre-ART group (*n* = 8 macaques), black circles/lines represent the control group (*n* = 5 macaques). In **f** and **g**: the horizontal bar in each group represents the mean. A two-sided Welch’s *t*-test was used to compare values between the groups. *P < 0.05, **P < 0.01, ***P < 0.001, ****P < 0.0001. Source data

In the current study, 21 Indian-origin, adult RM (Extended Data Table 1) were infected intravenously (i.v.) with 10,000 IU of barcoded SIV_mac239M_ containing ~10,000 clonotypes at approximately equal proportions^18^, and a standard ART regimen (Tenofovir/TDF, Emtricitabine/FTC, Dolutegravir/DTG) was started at day 14 post-infection (p.i.). The RMs were divided into three groups (Fig. 1a): (1) 8 animals received a single 50 mg kg^−1^ dose of the anti-CD8α-depleting antibody, MT807R1, 1 d before SIV infection (Group-1: Pre-infection CD8^+^ depletion); (2) 8 animals received MT807R1 1 d before ART initiation (Group-2: Pre-ART CD8^+^ depletion); and (3) 5 animals served as controls (Group-3). All RMs were treated with ART for 50 weeks and then underwent analytical treatment interruption (ATI), after which they were monitored for 12 weeks before necropsy.

**Extended Data Fig. 1:**
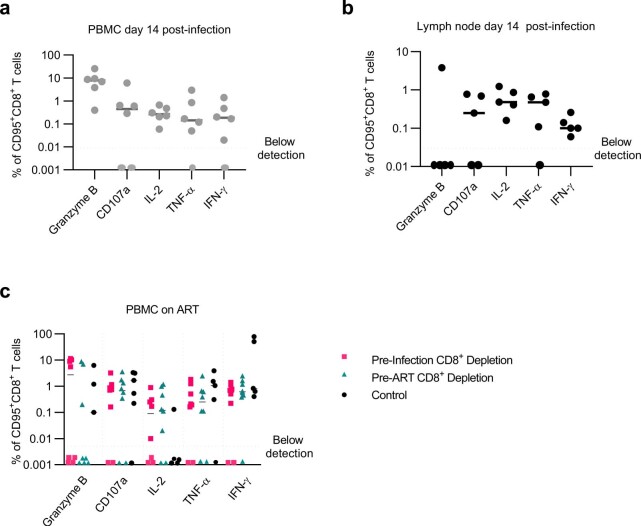
SIV-specific CD8^+^ T cell responses 14 days post-infection and during long-term ART. Frequency of CD107a^+^, TNF-α^+^, IFN-γ^+^, IL-2^+^, and Granzyme B^+^ cells was measured in memory CD8^+^ T cells from peripheral blood (**a**; n = 6 macaques, grey circle), lymph node (**b**; n = 5 macaques, black circle) at day 14 post-infection, and from peripheral blood during long-term ART (**c**). For Figure **c**: Pre-infection depletion group (n = 8 macaques, magenta square), Pre-ART depletion group (n = 8 macaques, teal triangle), and control group (n = 5 macaques, black circle). Horizontal dashed line represents the below detection limit.

**Extended Data Fig. 2:**
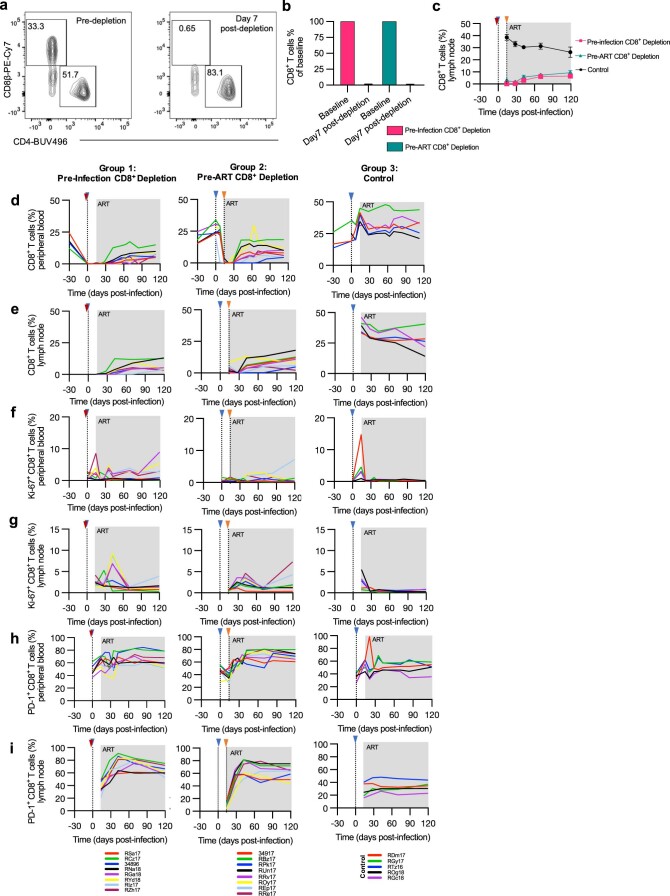
Effectiveness of CD8^+^ T cell depletion, longitudinal CD8^+^ T cell frequencies, Ki-67, and PD-1 expression. **a**. Representative flow cytometry shows the absence of CD8β^+^ cells 7 days post-depletion in the peripheral blood. **b**. Comparison of CD8β^+^ frequency to pre-depletion baseline calculated in all CD8^−^depleted macaques (n = 16). For figures **c**–**i**: Blue triangle indicates SIVmac239M infection, red triangle indicates CD8^+^ T cell depletion prior to infection, orange triangle indicates CD8^+^ T cell depletion prior to ART initiation. Grey box represents ART. **c**. Lymph node CD8^+^ T cell kinetics in Pre-infection CD8^+^ depletion group (n = 8 macaques, magenta line), Pre-ART CD8^+^ depletion group (n = 8 macaques, teal line), and control group (n = 5 macaques, black line). Data are mean ± s.e.m. In Figures **c**–**i:** lines on each graph represents individual animals in each study group and indicated in key under each column of graphs. **d, e**. Longitudinal CD8^+^ T cell percentage in peripheral blood (**d**), lymph node biopsies (**e**) in the three experimental groups. **f,g**. Longitudinal flow cytometry analysis of Ki-67 expression in bulk CD8^+^ T cells after CD8^+^ T cell depletion in peripheral blood (**f**) and lymph node biopsies (**g**). **h, i**. Longitudinal flow cytometry analysis of PD-1 expression in bulk CD8^+^ T cells after CD8^+^ T cell depletion in peripheral blood and lymph node biopsies.

**Extended Data Fig. 3:**
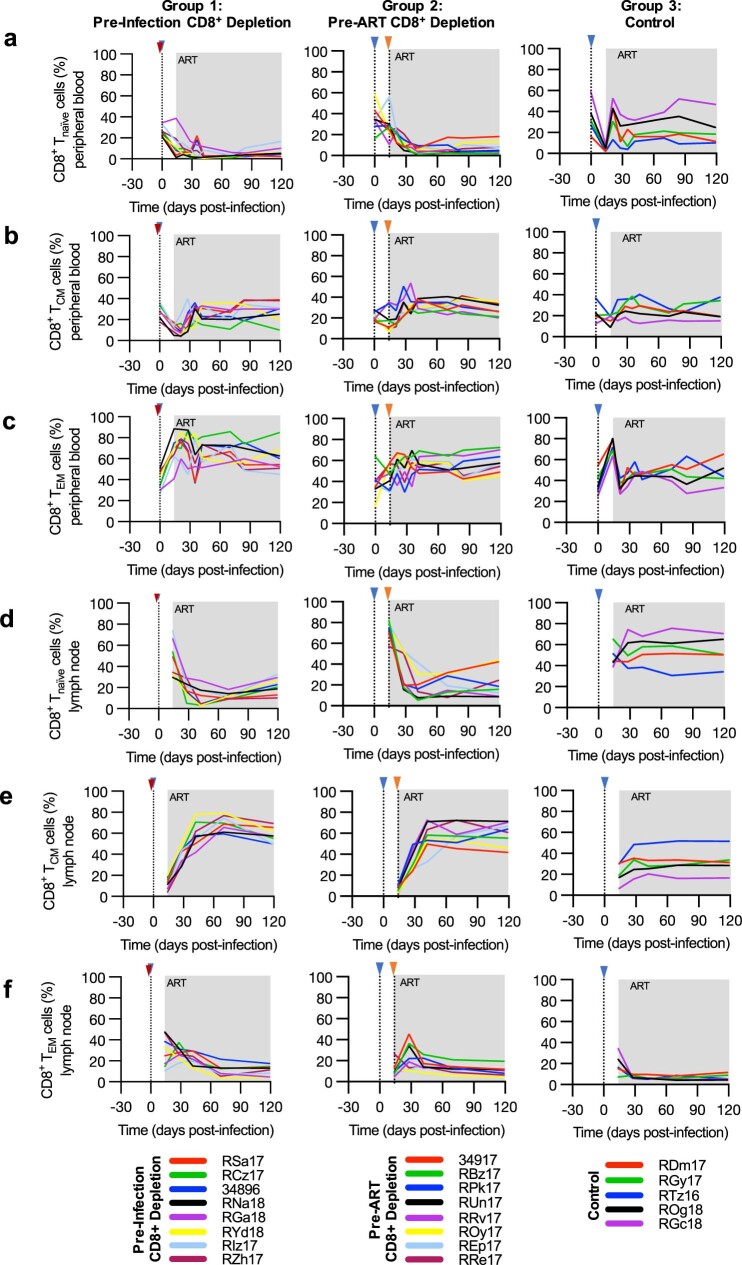
Phenotypic changes to CD8^+^ T cells after depletion and reconstitution. Longitudinal flow cytometry analysis after CD8^+^ T cell depletion in Pre-infection CD8^+^ depletion group (n = 8 macaques), Pre-ART CD8^+^ depletion group (n = 8 macaques), and control group (n = 5 macaques). Blue triangle indicates SIVmac239M infection, red triangle indicates CD8^+^ T cell depletion prior to infection, orange triangle indicates CD8^+^ T cell depletion prior to ART initiation. Grey box represents ART. Lines on each graph represents individual animals in each study group and indicated in key under each column of graphs. Percentage of CD28 + CD95^−^ T_N_, CD28^+^CD95^+^ T_CM_, and CD28^−^CD95^+^ T_EM_ CD8^+^ T cells in peripheral blood (**a-c**) and lymph nodes (**d**–**f**).

**Extended Data Fig. 4:**
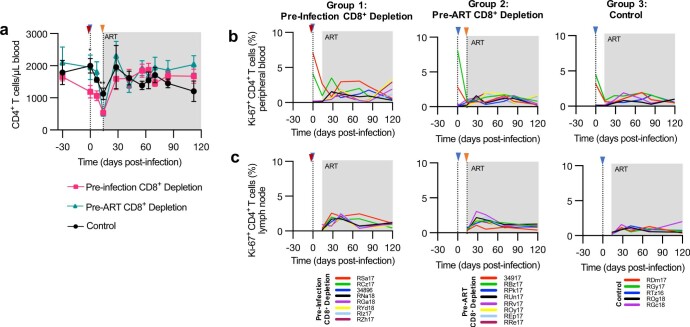
CD8^+^ T cell depletion in SIV-infected Rhesus macaques does not increase CD4^+^ T cell proliferation. In Figures **a–c**: Blue triangle indicates SIVmac239M infection, red triangle indicates CD8^+^ T cell depletion prior to infection, orange triangle indicates CD8^+^ T cell depletion prior to ART initiation. Grey box represents ART. **a**. CD4^+^ T cell count in peripheral blood in Pre-infection CD8^+^ depletion group (n = 8 macaques, magenta line), Pre-ART CD8^+^ depletion group (n = 8 macaques, teal line), and control group (n = 5 macaques, black line). Data are mean ± s.e.m. Kruskal Wallis test was used to compare the value between the groups. **b,c**. Lines on each graph represents individual animals in each study group and indicated in key under each column of graphs. Longitudinal flow cytometry analysis after CD8^+^ T cell depletion Pre-infection CD8^+^ depletion group (n = 8 macaques, magenta line), Pre-ART CD8^+^ depletion group (n = 8 macaques, teal line), and control group (n = 5 macaques, black line). Percentage of bulk CD4^+^ T cells that express Ki-67 in peripheral blood (**b**) and lymph nodes (**c**). * p value less than 0.05; ** p value less than 0.01.

**Extended Data Fig. 5:**
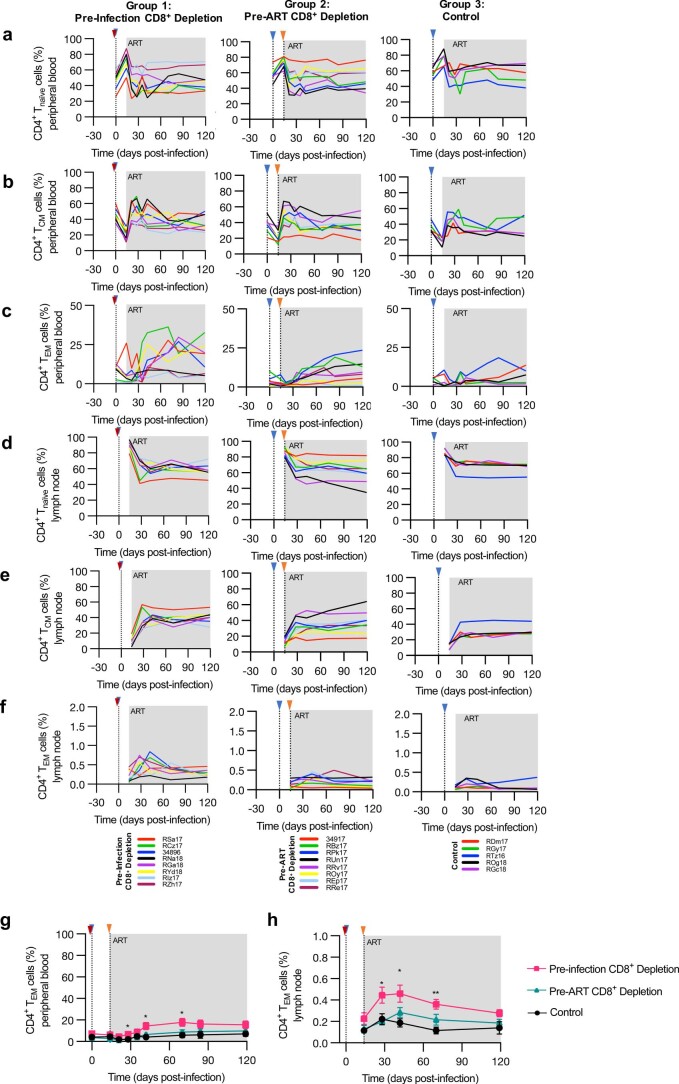
Phenotypic changes to CD4^+^ T cells after CD8^+^ depletion. Longitudinal flow cytometry analysis after CD8^+^ T cell depletion in Pre-infection CD8^+^ depletion group (n = 8 macaques, magenta line), Pre-ART CD8^+^ depletion group (n = 8 macaques, teal line), and control group (n = 5 macaques, black line). Blue triangle indicates SIVmac239M infection, red triangle indicates CD8^+^ T cell depletion prior to infection, orange triangle indicates CD8^+^ T cell depletion prior to ART initiation. Grey box represents ART. Lines on each graph represents individual animals in each study group and indicated in key under each column of graphs. Percentage of CD28^+^CD95^-^ T_N_, CD28^+^CD95^+^ T_CM_, and CD28^−^CD95^+^ T_EM_ CD4^+^ T cells in peripheral blood (**a-c,g**) and lymph nodes (**d-f,h**). For Figures **g, h:** Data are mean ± s.e.m. Kruskal-Wallis test was used to compare values between the groups. * p value less than 0.05; ** p value less than 0.01.

The decisions to start ART at day 14 p.i. and to deplete CD8^+^ lymphocytes at day 13 p.i. in the pre-ART CD8^+^ depletion group were based on previous studies that identified the emergence of SIV-specific CD8 T cells and the presence of CTL pressure (as measured by escape mutants in the prevailing virus quasi-species) by 14 d p.i.^11,19^. In the current study, we quantified SIV-specific CD8^+^ T cells at day 14 p.i. in the undepleted group by using ex vivo stimulation with SIV peptides and measured the frequency of CD8^+^CD95^+^ T cells expressing Granzyme B, CD107a, IL-2, TNF-α and IFN-γ in response to these peptides (Extended data Fig. 1a,b). As expected, when examining CD8^+^ T cells collected from lymph nodes (LN) of undepleted RMs as well as peripheral blood mononuclear cells (PBMCs) from a previous study also using SIV_mac239_, we found that robust SIV-specific CD8^+^ T-cell responses are present at day 14 p.i. in all animals. As such, these data confirm that the relatively short window of in vivo antigen exposure (that is, day 0–14 p.i.) is sufficient to generate virus-specific CD8^+^ T-cell responses. In line with previous studies^16,17,20^, treatment with MT807R1 was followed by a rapid depletion of 99.7–99.8% of CD8^+^ T cells in the blood in the pre-infection and pre-ART CD8^+^ depletion groups compared with the baseline (Fig. 1b and Extended data Fig. 2a,b). In LN, where a baseline sample was not available, the levels of CD8^+^ T cells post MT807R1 administration were 99.6% and 95.3% in the pre-infection and pre-ART CD8^+^ depletion groups, respectively, as compared with undepleted animals (Extended Data Fig. 2c). CD8^+^ lymphocyte depletion was followed by repopulation of these cells by day 42 after MT807R1 administration (Extended Data Fig. 2d,e). As expected, reconstitution of CD8^+^ T cells was associated with increased frequencies of CD8^+^ T cells expressing Ki-67 and PD-1 in both blood and LN (Fig. 1c,d and Extended Data Fig. 2f–i). As shown in Extended Data Fig. 3a–f, the majority of repopulating CD8^+^ T cells were included in the CD28^+^CD95^+^ central memory (T_CM_) or the CD28^−^CD95^+^ effector memory (T_EM_) subpopulations, with slower reconstitution of the CD28^+^CD95^−^ naïve cells (T_N_). Of note, CD8^+^ lymphocyte depletion was not associated with major changes in the levels of circulating CD4^+^ T cells as compared with undepleted animals (Extended Data Fig. 4a). Similarly, we did not observe any significant change across groups in the fraction of circulating or LN-based CD4^+^ T cells expressing Ki-67 (Extended Data Fig. 4b,c), or in the fraction of circulating or LN-based CD4^+^ T_N_ or T_CM_ cells (Extended Data Fig. 5a–f). In contrast, a moderate but significant increase in the fraction of CD4^+^ T_EM_ was observed in both blood and LN of RMs belonging to the ‘pre-infection’ CD8^+^ depletion group (Extended Data Fig. 5c,f–h).

**Extended Data Fig. 6:**
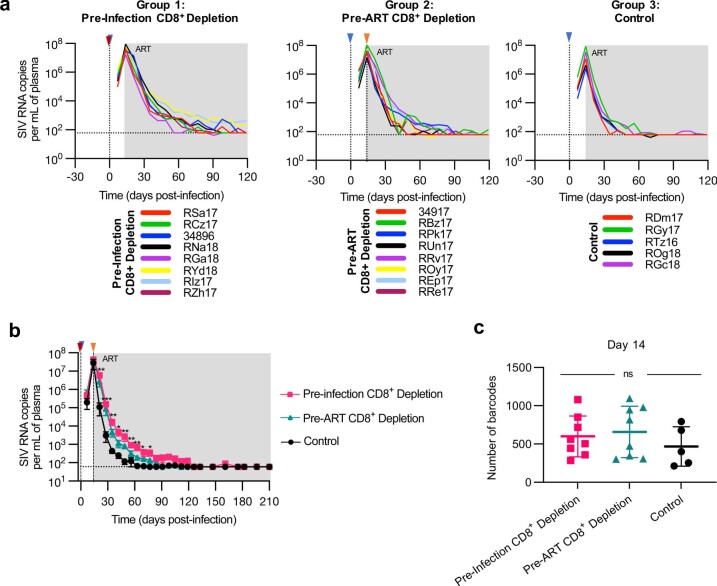
Longitudinal SIV plasma viremia and barcode analysis. For Figures **a, b**: SIV plasma viral load in the three experimental groups. Limit of detection is 60 copies of SIV RNA per mL of plasma (horizontal dashed line). Blue triangle indicates SIVmac239M infection, red triangle indicates CD8^+^ T cell depletion prior to infection, orange triangle indicates CD8^+^ T cell depletion prior to ART initiation. Grey box represents ART. **a**. SIV plasma viremia during the first 120 days p.i in the three experimental groups. Lines on each graph represents individual animals in each study group and indicated in key under each column of graphs. For Figures **b, c:** magenta squares/lines represent the Pre-Infection group (n = 8 macaques), teal triangles/lines represent the Pre-ART group (n = 8 macaques), black circles/lines represent the control group (n = 5 macaques). **b**. SIV plasma viremia until day 210 p.i in the three experimental groups. Data are mean ± s.e.m. Kruskal-Wallis tests were used to compare values between the groups**. c**. Analysis of the barcode diversity at day 14 post infection (at time of ART initiation). Data are mean ± SD. Kruskal-Wallis test was used to compare values between the groups. * p value less than 0.05; ** p value less than 0.01; *** p value less than 0.001; **** p value less than 0.0001.

At the time of ART initiation (day 14 p.i.), all RMs showed similar plasma viremia (Fig. 1e and Extended Data Fig. 6a,b), consistent with a previous study^21^, and a similar range of barcode diversity (Extended Data Fig. 6c). However, longitudinal quantitative measurement of viremia during the first 3 weeks of ART revealed a slower decline in RMs of Groups 1–2 (that is, pre-infection and pre-ART CD8^+^ depletion) (Fig. 1e), as compared with controls. In particular, we found that viremia at days 21, 28 and 35 p.i (that is, days 7, 14 and 21 from ART initiation) was significantly higher in the CD8^+^ depleted groups as compared with controls (Fig. 1f). Similarly, the viral load 7 d decline rates between both days 0 and 7 post-ART initiation and days 14 and 21 post-ART initiation were significantly lower in the CD8^+^ depleted groups as compared with controls (Fig. 1g). This slower decline of viremia notwithstanding, all RMs reached full virological suppression within 5 months of ART, with viremia below 60 copies per ml of plasma (Extended Data Fig. 6b).

Classic studies of viral dynamics following ART demonstrated a biphasic decay of viremia^22–24^. Most of the plasma virus is produced by short-lived productively infected cells (*t*_1/2_ ∼ 1 d), while a second population with a half-life on the order of weeks makes a smaller contribution to viremia. Assuming that ART was equally effective in suppressing de novo rounds of SIV replication in all groups, the observation of a significantly slower decline of viremia after ART in CD8^+^ depleted RMs indicates that, in the absence of CD8^+^ T cells, the in vivo lifespan of productively infected cells that produce most of the plasma virus is longer and/or the number of virions produced per infected cell is higher, presumably due to the experimental removal of SIV-specific CTL activity.

### Impact of CD8^+^ T-cell depletion on SIV-DNA^+^ CD4^+^ T cells

**Fig. 2:**
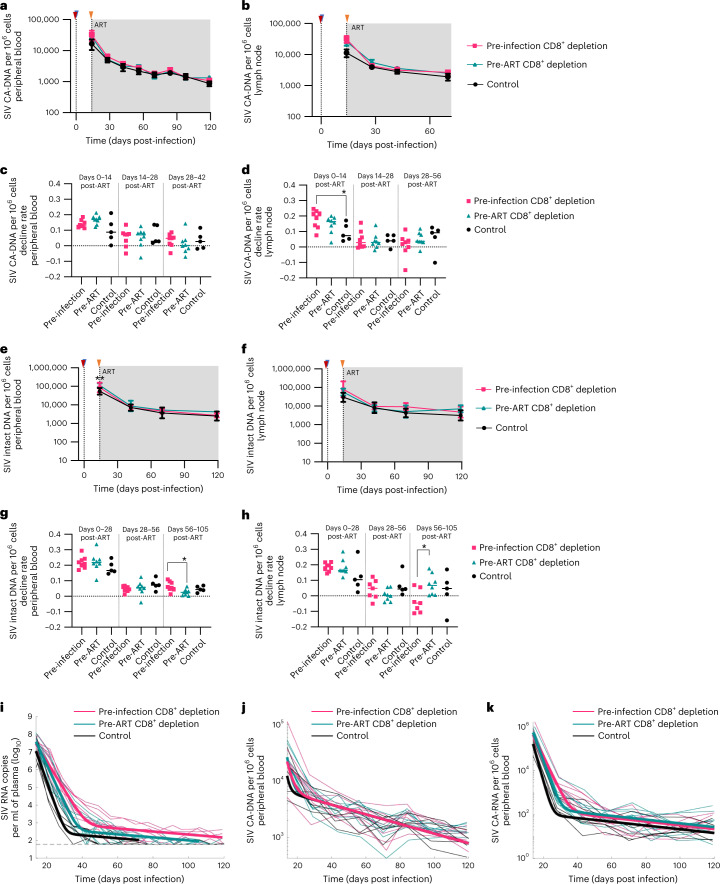
CD8^+^ T lymphocyte depletion does not increase the size of the SIV reservoir. **a**,**b**, SIV cell-associated DNA in peripheral blood (**a**) and in lymph nodes (**b**). **c**,**d**, CA-DNA decline rate between various time points after ART initiation in both peripheral blood (days 0 and 14; days 14 and 28; days 28 and 42) (**c**) and lymph nodes (days 0 and 14; days 14 and 28; days 28 and 56) (**d**). **e**,**f**, SIV intact proviral DNA in peripheral blood (**e**) and lymph nodes (**f**). **g**,**h**, Intact proviral DNA decline rate between various time points after ART initiation in both peripheral blood (days 0 and 28; days 28 and 56; days 56 and 105) (**g**) and lymph nodes (days 0 and 28; days 28 and 56; days 56 and 105) (**h**). **i**–**k**, Model fitting of the levels of SIV plasma viral load (**i**), CA-DNA (**j**) and CA-RNA (**k**). The group-based model predictions are shown as thick lines. The data for individual animals are shown as thin lines. The grey dashed line in **i** indicates the limit of detection. For each group, the parameters (Supplementary Table 2) were estimated under a nonlinear mixed-effects modelling approach. In **a**–**k**: Pre-infection depletion group (*n* = 8 macaques, magenta squares/line), Pre-ART group (*n* = 8 macaques, teal triangles/line) and control group (*n* = 5 macaques, black circles/line). In **a**, **b**, **e** and **f**: blue triangle indicates SIVmac239M infection, red triangle indicates CD8^+^ T-cell depletion before infection, and orange triangle indicates CD8^+^ T-cell depletion before ART initiation. Grey box represents ART. Longitudinal analysis after CD8^+^ T-cell depletion was performed in CD4^+^ T cells derived from peripheral blood and lymph node biopsies. Data are mean ± s.e.m. Kruskal Wallis test was used to compare the values between the groups. In **c**, **d**, **g** and **h**: the horizontal bar in each group represents the mean. A two-sided Welch’s *t*-test was used to compare the values between the groups. *P < 0.05, **P < 0.01, ***P < 0.001, ****P < 0.0001.

**Extended Data Fig. 7:**
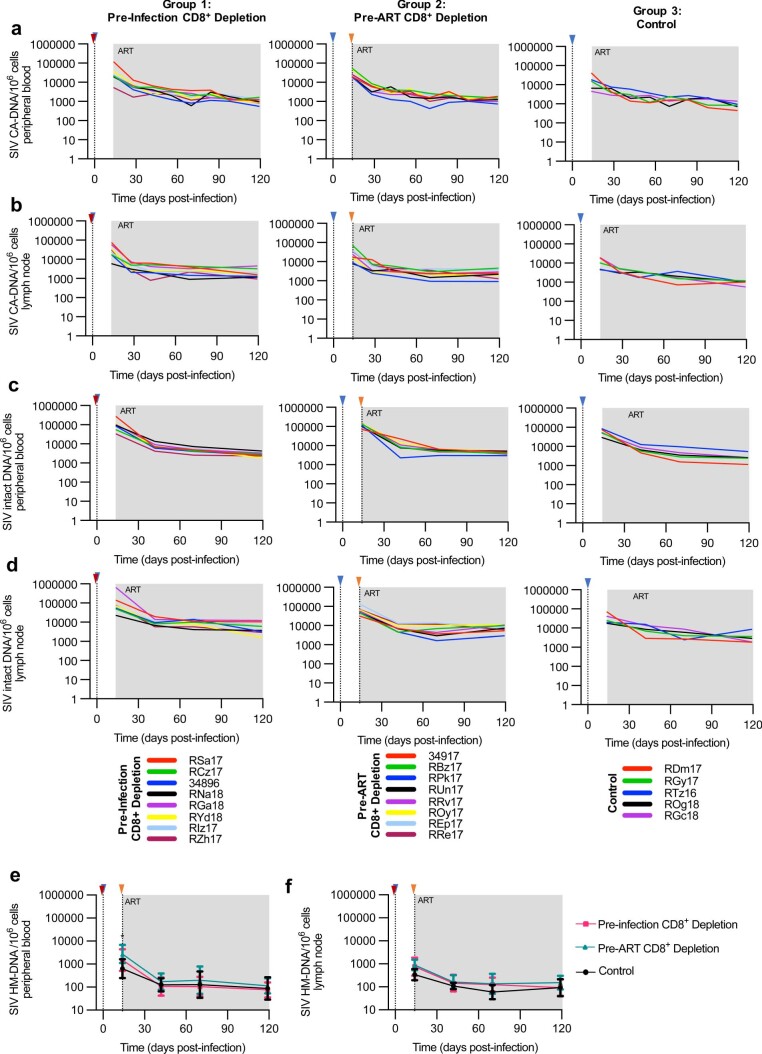
Longitudinal cell-associated DNA and intact proviral DNA. Longitudinal analysis after CD8^+^ T cell depletion in Pre-infection CD8^+^ depletion group (n = 8 macaques, magenta line), Pre-ART CD8^+^ depletion group (n = 8 macaques, teal line), and control group (n = 5 macaques, black line). Blue triangle indicates SIVmac239M infection, red triangle indicates CD8^+^ T cell depletion prior to infection, orange triangle indicates CD8^+^ T cell depletion prior to ART initiation. Grey box represents ART. Lines on each graph represents individual animals in each study group and indicated in key under each column of graphs. Cell-associated SIV DNA and intact proviral DNA were determined in CD4^+^ T cells derived from peripheral blood (**a, c**), and in lymph nodes (**b**, **d**). **e**, **f**. SIV hypermutated (HM) DNA were determined in CD4^+^ T cells derived from peripheral blood (**e**) and lymph node biopsies (**f**). Data are mean ± s.e.m. Kruskal-Wallis tests were used to compare values between the groups. * p value less than 0.05.

To determine the impact of CD8^+^ depletion on the size of the viral reservoir under ART, we next measured the levels of total cell-associated (CA) SIV-DNA in blood- and LN-derived CD4^+^ T cells over the first 4 months of treatment. As shown in Fig. 2a,b and Extended Data Fig. 7a,b, depletion of CD8^+^ T cells did not change the frequency of total CA-DNA^+^ cells at day 14 p.i. Importantly, the frequency of SIV-DNA^+^ cells declined after ART with similar kinetics in all groups of RMs, and converged to similar levels by day 116 and day 56 of ART in blood and LN, respectively (Fig. 2a,b and Extended data Fig. 7a,b). We next measured the decline rate of CA SIV-DNA^+^ CD4^+^ T cells between various time points on ART in both blood (days 0 and 14; days 14 and 28; days 28 and 42) and LN (days 0 and 14; days 14 and 28; days 28 and 56) and found no differences between groups, except for the isolated finding of a faster decline between days 0 and 14 in the LN of the pre-infection depletion group as compared with controls (Fig. 2c,d).

To next determine the impact of CD8^+^ depletion on the fraction of CD4^+^ T cells harbouring replication-competent virus, we used an SIV-adapted version of the recently described Intact Provirus DNA Assay (IPDA)^25^. This assay represents a reliable surrogate for the direct measurement of cells harbouring replication-competent virus by quantitative-viral outgrowth assay^25^, which was not possible to perform due to the small number of collected cells. As shown in Fig. 2e,f and Extended Data Fig. 7c,d, the longitudinal assessment of the frequency of IPDA^+^ CD4^+^ T cells in both blood and LN revealed similar kinetics between the two groups of CD8^+^ depleted RMs and controls. In addition, we compared the decline rate of SIV intact DNA copies per 10^6^ cells between various time points after ART in both blood (days 0 and 28; days 28 and 56; days 56 and 105) and LN (days 0 and 28; days 28 and 56; days 56 and 105) and again found no major differences between the three groups, except for a faster decline between days 56 and 105 in the blood of the pre-infection depletion group and in the lymph nodes of the pre-ART depletion group (Fig. 2g,h). In addition, we measured, at the same time points, the fraction of CD4^+^ T cells expressing hypermutated proviruses using the recently validated Hypermutated Provirus DNA Assay (HPDA^26^) and again found no significant differences in the kinetics of ART-induced decline of HPDA^+^ cells in the three groups (Extended Data Fig. 7e,f). Overall, these data indicate that CD8^+^ depletion performed either pre-infection or pre-ART does not change the decay kinetics of infected cells in blood or LN.

**Extended Data Fig. 8:**
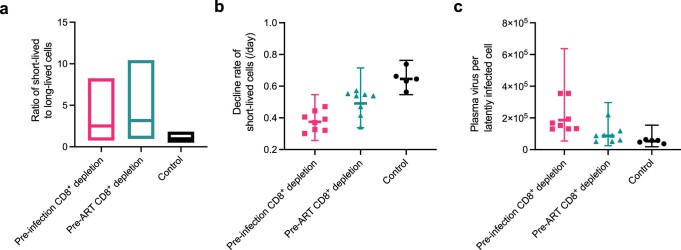
Mixed-effect model. For Figures **a–c**: magenta squares/lines represent the Pre-Infection group (n = 8 macaques), teal triangles/lines represent the Pre-ART group (n = 8 macaques), black circles/lines represent the control group (n = 5 macaques). **a**. Model estimates of the ratio of short-lived cells to long-lived cells at ART initiation. For each group, the parameter estimate is presented as the center bar representing the estimate, and the bounds of the box representing the upper and lower limits of the 95% confidence interval. Pre-Infection group: 71.4% (95% CI = 43.2%, 89.2%); Pre-ART group: 76.0% (95% CI = 48.9%, 91.3%); control group: 48.7% (95% CI = 33.2%, 64.6%); p < 0.001 for comparison of control and each treated group. For Figures **b, c**: Parameter estimates are presented as fixed effect ± 95% confidence intervals of the fixed effect. **b**. Model estimates of the decline rate of short-lived cells. Control group = 0.65 /day (95% CI = 0.55, 0.76) versus Pre-infection depletion group = 0.38 /day (95% CI = 0.26, 0.55), (p < 0.001 vs control) and pre-ART depletion group = 0.49 /day (95% CI = 0.34, 0.72), (p = 0.010 vs. control) respectively. **c**. Model estimates of the plasma virus per long-lived cell. Control group = 5.2 × 10^4^ SIV RNA copies mL^−1^/(CA-DNA copy/10^6^ cells) (95% CI = 3.0 × 10^4^, 9.1 × 10^4^) versus Pre-infection = 1.86 × 10^5^ (95% CI = 5.4 × 10^4^, 6.4 × 10^5^) (p < 0.001 vs. control) and Pre-ART = 8.7 × 10^4^ (95% CI = 2.5 × 10^4^, 3.0 × 10^5^), (p = 0.14 vs. controls).

The analysis described above suggests that CD8^+^ lymphocyte depletion is associated with slower initial decline of viremia after ART. However, we observed in depleted and control macaques a similar early decline of the total number of SIV-DNA^+^ CD4^+^ T cells and no long-term effect on either SIV-DNA^+^ CD4^+^ T cells or IPDA^+^ and HPDA^+^ CD4^+^ T cells. A potential explanation is that short-lived cells that produce most of the plasma virus represent only a fraction of the circulating SIV-DNA, which also includes long-lived cells^27^. Thus, a more rapid loss of short-lived cells can be more difficult to detect above the high proportion of long-lived SIV-DNA^+^ cells. Moreover, the majority of infected cells and the major source of plasma virus is probably in tissues such as LN and gut-associated lymphoid tissue, which is a major site of viral replication and CD4^+^ T cell depletion in the SIV model^28^. To better quantify the virus dynamics after ART, we developed a mathematical model that incorporates a population of short-lived productively infected cells and a second population of long-lived latently infected cells. The model incorporated different lifespans for these populations, as well as different per-cell viral production and differential sensitivity to CD8-mediated CTL targeting. We fitted this model to the combined data on viremia, CA-RNA and CA-DNA over time to estimate the effects of CD8^+^ T cells on productively and latently infected cells (Fig. 2i–k and Extended Data Fig. 8a–c). This model reproduces the expected two-phase kinetics of viral decay after treatment, with the first phase representing rapid loss of short-lived cells producing high levels of virus. Fitting of the model parameters shows three significant changes in infection dynamics as a result of CD8^+^ depletion. First, CD8^+^ depletion affects infection dynamics in early infection, leading to a higher fraction of short-lived productively infected cells at the time of treatment (48.7% for control group, 71.4% and 76.0% for pre-infection and pre-ART depletion, respectively; *P* < 0.001 for comparison of control and each treated group) (Extended Data Fig. 8a). In addition, CD8^+^ T-cell depletion was associated with a slower rate of death of productively infected cells (controls, 0.65 d^−1^ vs pre-infection depletion, 0.38 d^−1^ (*P* < 0.001 vs control) and pre-ART depletion, 0.49 d^−1^ (*P* = 0.010 vs control)) (Extended Data Fig. 8b). In addition, CD8 depletion led to a higher level of average plasma virus production per long-lived infected cell in pre-infection depleted animals (controls, 5.2 × 10^4^ SIV RNA copies ml^−1^/CA-DNA copies per 10^6^ cells vs pre-infection, 1.86 × 10^5^ (*P* < 0.001 vs control) and pre-ART, 8.7 × 10^4^ (*P* = 0.14 vs controls)) (Extended Data Fig. 8c).

### CD8^+^ depletion does not impact the virus rebound after ATI

**Fig. 3:**
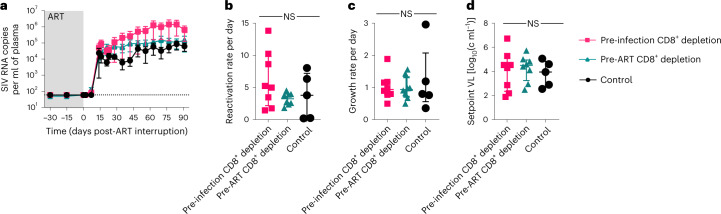
Viral rebound after treatment interruption. **a**, SIV plasma viral load after treatment interruption until necropsy in the three experimental groups. Limit of detection is 60 copies of SIV RNA per ml of plasma (horizontal dotted line). Grey box represents ART. Data are mean ± s.e.m. Kruskal Wallis test was used to compare the values between the groups. **b**, Frequency of virus reactivation after treatment interruption (estimated on the basis of the number and size of barcoded clonotypes observed). **c**, Viral load growth rate per day during early rebound. **d**, Setpoint viral load (time-weighted area under curve of viral load at days 30–60 after viral detection). In **a**–**d**: Pre-infection depletion group (*n* = 8 macaques, magenta square/line), Pre-ART depletion group (*n* = 8 macaques, teal triangle/line) and control group (*n* = 5 macaques, black circle/line). In **b**–**d**: bars indicate the median with interquartile range. Kruskal Wallis test was used to compare the values between the groups.

**Extended Data Fig. 9:**
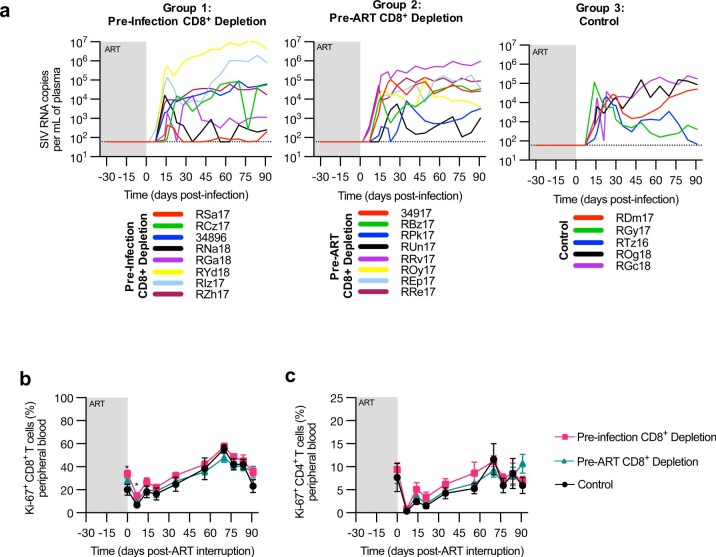
Longitudinal SIV plasma viremia and phenotypic changes after treatment interruption. **a**. Longitudinal viral loads in the three experimental groups after treatment interruption. Limit of detection is 60 copies of SIV RNA per mL of plasma (horizontal dashed line). Grey box represents ART. Lines on each graph represents individual animals in each study group and indicated in key under each column of graphs. **b,c**. Longitudinal flow cytometry analysis after treatment interruption in Pre-infection CD8 + depletion group (n = 8 macaques, magenta line), Pre-ART CD8 + depletion group (n = 8 macaques, teal line), and control group (n = 5 macaques, black line). Grey box represents ART. Percentage of bulk CD8^+^ (**b**) or CD4^+^ (**c**) T cells that express Ki-67 in peripheral blood. Data are mean ± s.e.m. Kruskal-Wallis tests were used to compare values between the groups. * p value less than 0.05.

Most of the infected cells detected at ART initiation and during the phase of initial decay do not become part of the stable latent reservoir^27^. In the current study, we detected no clear effect of CD8^+^ depletion on this population. As a functional assessment of the impact of CD8^+^ depletion on the reservoir size, we next performed an ATI at week 50 after ART initiation, that is, when viremia was undetectable in all treated animals (Extended Data Fig. 9a). This is the most robust way to evaluate the persistence of a clinically significant population of latently infected cells. As shown in Fig. 3a, all RMs experienced a rebound of viremia within 15 d from ATI, with no animals showing control of viremia (Extended Data Fig. 9a). While the area under the curve for viremia was not significantly different between groups, we noted a trend towards increased virus replication post-ATI in the RMs belonging to group-1 (that is, pre-infection CD8^+^ depletion). To determine whether this trend could be attributed to a paucity of SIV-specific CD8^+^ T cells, we assessed the frequency of CD8^+^CD95^+^ T cells expressing Granzyme B, CD107a, IL-2, TNF-α and IFN-γ in response to ex vivo stimulation with SIV peptides on PBMCs collected during ART at weeks 17–19 p.i. (Extended Data Fig. 1c). We found similar frequencies of SIV-specific CD8^+^ T-cell responses between the CD8^+^ depleted groups and the controls, suggesting that the antigen exposure after CD8^+^ T-cell reconsitution is sufficient to generate SIV-peptide specific responses even while the animals are on ART. As expected, the higher levels of viremia observed after ATI were associated with a detectable increase in both CD4^+^ and CD8^+^ T-cell activation, as measured by the fraction of Ki-67^+^ cells (Extended Data Fig. 9b,c). To quantitatively determine whether and to what extent the CD8^+^ depletion impacts the rebound of viremia after ATI, we used a previously described method that integrates the ratio of the number of copies of different barcodes and the growth rate of virus^18,29^. Consistent with the similar kinetics of viral rebound, we found a comparable reactivation rate (Fig. 3b) and growth rate per day of viremia (Fig. 3c) among all groups. In addition, we found that the setpoint viral load was not statistically different among the groups (Fig. 3d). Collectively, these data indicate that the analysis of the kinetics of virus rebound after ATI as a functional assessment of the overall size of the reservoir of replication-competent virus failed to reveal any statistically significant difference between depleted and undepleted animals. As such, this analysis confirms that CD8^+^ depletion conducted either before SIV infection or immediately before ART initiation does not lead to an expanded reservoir.

## Discussion

The current study experimentally measures the role of CD8^+^ lymphocytes on the establishment of the persistent reservoir under ART. The study involved CD8^+^ depletion either before SIV infection or immediately before ART initiation, which was conducted at day 14 p.i. The main results of the study are that CD8^+^ depletion (1) is associated with longer lifespan of productively infected cells and/or increased virus production on a per-cell basis, (2) does not substantially increase the size of the infected cell population in blood or LN as measured by the total SIV-DNA^+^ CD4^+^ T cells and cells harbouring intact proviruses and (3) does not substantially increase the size of the persistent reservoir as assessed functionally through analysis of virus rebound after ATI. We believe that these results represent a major advance in our understanding of the mechanisms responsible for the establishment of the persistent reservoir under ART.

Two previous studies involving CD8^+^ depletion in SIV-infected RMs at ART initiation indicated that CD8^+^ T cells do not reduce the lifespan of productively infected cells^30,31^; however, in these studies, ART was started during chronic SIV infection, that is, when the efficacy of CTLs is already impaired by viral escape and exhaustion. In the current experiment, ART was initiated during acute infection, which represents the time of peak efficacy for virus-specific CTL activity^19,32–34^. Consistent with this paradigm, we observed a clear effect of CD8^+^ depletion on the slope of viremia decline, which we postulate to be related primarily to the ablation of SIV-specific CTLs, which are known to reduce virus replication during acute HIV/SIV infections (reviewed in ref. ^5^). Of note, the alternative possibility that the slower decline of viremia observed in the CD8^+^ depleted groups was due to increased numbers of virus producing ‘activated/proliferating’ CD4^+^ T cells (either due to homoeostatic stimuli or in response to higher antigen levels) was not supported by the measurement of CD4^+^Ki-67^+^ T cells. In this study, the impact of CD8 depletion on the prevailing level of CD4^+^ T-cell activation appears less dramatic than in previous studies^15,20,35^. This partial discrepancy may be explained by the fact that in the current experiment, the RMs underwent CD8 depletion at a time when CD4^+^ T-cell activation is also influenced by both acute SIV infection and ART initiation, thus possibly diluting the direct effect of CD8^+^ depletion on CD4^+^ T-cell activation.

Evidence in favour of a major role for CTLs during acute HIV/SIV infection includes the observations that (1) virus-specific CD8^+^ T-cell responses expand during the decline of post-peak plasma viremia^6,7^; (2) virus CTL escape mutations emerge in response to CD8^+^ T-cell pressure^8–11^; (3) specific MHC-I alleles are associated with slower disease progression^36,37^; and (4) elite controller phenotype is associated with CTLs with polyfunctionality, proliferative capacity and in vitro killing potential^38–41^. As such, the formal demonstration that the removal of CD8^+^ T-cell-mediated CTL activity during acute SIV infection results in longer lifespan of productively infected cells and/or increased virus production on a per-cell basis fits nicely with a large body of experimental evidence on the antiviral role of CTLs.

However, a strikingly unexpected finding of the current study is that CD8^+^ depletion did not induce a significant increase in the size of the persistent latent reservoir as assessed either directly or via kinetic analysis of viremia rebound after ATI. There are several, non-mutually exclusive hypotheses to explain this observation: (1) the bulk of cells that are killed by CTL are destined to die anyway by either virus-mediated cytopathic effect and/or activation-induced cell death; (2) the majority of cells that form the persisting reservoir do not experience sufficient levels of active virus production to be targeted by CTL activity; and (3) the majority of cells that form the persisting reservoir are not targeted by CTLs because of specific anatomic/histological locations and/or specific phenotypic changes (that is, class-I downmodulation). While the current experiment did not address the contribution of each of these potential events, it is remarkable how this study has revealed a clear disconnection between a powerful role of CD8^+^ mediated CTLs in controlling virus production and a largely ineffective role in preventing the establishment of the reservoir under ART. Of note, the current experiment did not address the possibility that such an anti-reservoir effect of CTLs may be achieved through vaccination prior to infection or use of check-point inhibitors, which will be the topic of further studies.

Although all animals were durably suppressed at the time of ATI, it is formally possible that some level of residual (that is, <60 copies per ml) and/or transient (that is, at times when sampling was not conducted) viremia was present under ART, as shown for HIV infection^42–44^. In this case, residual virus production and/or replication could have also contributed to the observed stability of the virus reservoir.

In a series of recent studies, we demonstrated that in addition to antigen-specific, MHC-class-I-restricted CTL, CD8^+^ T cells suppress HIV/SIV production through a non-cytolytic, non-MHC-I-restricted mechanism resulting in potent inhibition of virus transcription^14,16^. The role played by the CD8^+^ T-cell-mediated transcriptional suppression of SIV in the current experiment is unclear, as we have neither formally proved that this effect is present in vivo during the early stages of infection, nor have we been able to quantify its role in suppressing virus replication as compared to canonical CTL activity. Theoretically, it is possible that the removal of CD8^+^ T-cell-mediated transcriptional suppression contributed to the prolonged detection of productively SIV-infected cells by promoting active virus transcription and thus synergizing with the lifespan increase caused by CTL removal. Even in this scenario, however, the effect of removing the CD8^+^ T-cell-mediated suppression of SIV transcription would be delaying but not suppressing the establishment of the persistent reservoir, unless this non-cytolytic activity results in a yet unreported pro-survival effect of productively SIV-infected cells. For this reason, the most parsimonious explanation of the key results of the current study is that the suppression of SIV transcription by CD8^+^ T cells is not a major determinant of the observed data.

The possibility that the persistent HIV/SIV reservoir is formed by cells that were never susceptible to CTL activity regardless of the responsible mechanisms (that is, direct latency formation, anatomic sanctuary, poor intrinsic CTL efficiency and so on) has important implications in terms of potential therapeutic approaches to eliminate this reservoir. In this regard, we are planning future experiments in which the dynamics of reservoir establishment using the current experimental approach (that is, CD8^+^ depletion and early ART initiation) will be studied in RMs that were vaccinated before SIV infection with CTL-eliciting vaccines. Further studies that aim to identify the specific molecular and cellular mechanisms by which the early establishment of the persistent reservoir under ART is resistant to CD8^+^ T-cell-mediated CTL activity may enable the development of novel, immune-based strategies that will substantially reduce the size of this reservoir in ART-treated HIV-infected individuals.

## Methods

### Ethics statement

All animal experimentations were conducted following guidelines established by the Animal Welfare Act and by the NIH’s Guide for the Care and Use of Laboratory Animals, 8th edition. All the procedures were approved by the Emory University Institutional Animal Care and Use Committee (Permit number YER2003384). Animal care facilities at Emory National Primate Research Center are accredited by the US Department of Agriculture and the Association for Assessment and Accreditation of Laboratory Animal Care International.

### Animals, SIV infection, CD8^+^ depletion and antiretroviral therapy

This study included 21 Indian RMs housed at Emory National Primate Research Center in Atlanta, Georgia (3 females, 18 males; 3–4 years at the start of the study).

All the animals were Mamu*B08^-^ and Mamu*B17^-^; the following macaques were Mamu*A01^+^: RDm17, RSa17, RTz16, 34917, RPk17, 34896, RUn17, ROg18, RGa18.

RMs were infected intravenously with 10,000 IU of barcoded SIVmac239M containing around 10,000 clonotypes present at approximately equal proportions^18^. The macaques were stratified in three groups (Fig. 1a): 8 macaques received one dose of the anti-CD8α-depleting antibody, MT807R1, at 50 mg kg^−1^ 1 d before the infection; 8 macaques received the same dose of MT807R1 1 d before starting ART; 5 macaques served as a control group. At 14 d p.i., a daily triple formulation antiretroviral therapy (ART) was initiated in all RMs, consisting of dolutegravir (DTG; 2.5 mg kg^−1^ d^−1^, provided by ViiV Pharmaceuticals), tenofovir disoproxil fumarate (TDF; 5.1 mg kg^−1^ d^−1^, provided by Gilead) and emtricitabine (FTC; 40 mg kg^−1^ d^−1^, provided by Gilead) that was maintained for up to 50 weeks before ATI. After ATI, all animals were monitored for 12 weeks before necropsy.

### Sample collection and processing of tissues

Peripheral blood (PB) and LN biopsies (axillary or inguinal region) were conducted longitudinally and at necropsy as previously described^45^. Briefly, blood samples were used for a complete blood count and routine chemical analysis, and plasma was separated by centrifugation within 1 h of phlebotomy. PBMCs were isolated from whole blood by density gradient centrifugation. For LN biopsies, the skin over the axillary or inguinal region was clipped and surgically prepped. An incision was then made in the skin over the LN, which was exposed by blunt dissection and excised over clamps. LNs were then homogenized and passed through a 70 um cell strainer to isolate lymphocytes. All samples were processed, fixed (1% paraformaldehyde) and analysed within 24 h of collection.

### Flow cytometry

Multiparametric flow cytometry was performed using standard procedures on PBMCs and mononuclear cells derived from LN biopsies using anti-human mAbs previously shown to be cross-reactive in RM^16,45–47^. The following antibodies were used at 37 °C for 30 min: CCR5-BV650 (1:200, clone 3A9; BD Biosciences, 564999) and CCR7 FITC (1:200, clone 150503; BD Biosciences, 561271), in addition to LIVE/DEAD aqua viability dye (1 μl of 1:20 PBS dilution, Thermo Fisher, L34966). The following antibodies were then used at room temperature for 30 min: CD3-APC-Cy7 (1:200, clone SP34-2; BD Biosciences, 557757), CD4-BUV496 (1:200, clone SK3; BD Biosciences, 564651), CD8α-BV711 (1:200, clone RPA-T8; Biolegend, 301044), CD8β-PE-Cy7 (1:200, clone SIDI8BEE; Invitrogen, 14-5273-82), CD45RA-Pe-Cy5 (1:200, clone 5H9; BD Biosciences, 552888), CD62L-BV786 (1:200, clone SK11; BD Biosciences, 565311), CD95-BV605 (1:200, clone DX2; Biolegend, 305628), PD-1-BV421 (1:200, clone EH12.2H7; Biolegend, 329920), CD14-BV510 (1:200, clone M5E2; Biolegend, 301842), CD20-BV510 (1:200, clone 2H7; Biolegend, 302340), NKG2A (also known as CD159a-APC) (1:200, clone Z199; Beckman Coulter, A60797), CD28-BUV737 (1:200, clone CD28.2; BD Biosciences, 612815), CD69-Pe-CF594 (1:200, clone FN50; BD Biosciences, 562617), CD25-BUV395 (1:200, clone 2A3; BD Biosciences, 564034) and HLA-DR-PerCP-Cy5.5 (1:200, clone G46-6; BD Biosciences, 552764). After fixation and permeabilization with Fixation/Permeablization kit (BD Biosciences), cells were stained with Ki-67-AF700 (1:200, clone B56; BD Biosciences, 561277) at room temperature for 30 min. All the antibodies were used following the manufacturers’ recommendations. Data acquisition was performed on an LSR II (BD Biosciences) driven by FACS DiVa software and analysed using FlowJo software (version 10.8; TreeStar).

### Plasma SIV RNA, cell-associated RNA and DNA

Plasma SIV viral loads were determined by standard quantitative RT–PCR with a sensitivity of 60 copies per ml as previously described^48^.

CD4^+^ T cells were enriched from PBMCs and mononuclear cells derived from lymph node biopsies using a CD4^+^ T cell non-human primate isolation kit (Miltenyi Biotech) on an LS column for all samples following the manufacturer’s specifications. Enriched cells were then aliquoted into 1 million or 5 million CD4^+^ T cells and lysed in 350 μl Buffer RLT Plus RNeasy Plus lysing buffer (Qiagen) with 1 mM 2-mercaptoethanol (Sigma-Aldrich). Total cell-associated SIVmac239M gag DNA and RNA were quantified as previously described^49,50^. Briefly, cell-associated SHIV RNA and DNA levels were measured simultaneously in total CD4^+^ T cells isolated from PBMCs and lymph nodes (1,000,000 to 5,000,000 cells) lysed in Buffer RLT Plus (Qiagen) plus 2-mercaptoethanol. Both DNA and RNA were extracted using the Allprep DNA/RNA mini kit (Qiagen). Quantification of SHIV *gag* DNA was performed on the extracted DNA by quantitative PCR using the 5′ nuclease (*Taq*Man) assay with an ABI7500 system (PerkinElmer). For cell number quantification, quantitative PCR was performed simultaneously with monkey albumin gene copy numbers. RNA was reverse transcribed using a high-capacity complementary DNA reverse transcription (RT) kit (Thermo Fisher) and random hexamers. SHIV *gag* and the rhesus macaque *CD4* gene were quantified by qPCR of the resultant cDNA using *Taq*Man universal master mix II (Thermo Fisher). Primer and probe sequences are detailed in the Supplementary Table 6.

### IPDA and HPDA

Genomic DNA was extracted with a QIAamp DNA mini kit (Qiagen) from cryopreserved CD4^+^ T cells enriched from PBMCs and lymph node mononuclear cells. IPDA and HPDA were performed as previously described^25,26^. Briefly, each sample was assayed in triplicate using three separate duplex reactions: the SIV IPDA, RPP30 and *env*-2LTRc assays. The IPDA measures intact proviruses using an amplicon in *pol* and one in *env*, with 2 labelled probes that detect intact proviruses and 2 unlabelled competition probes that exclude proviruses with G-to-A hypermutation. The RPP30 assay uses 2 amplicons in the host RPP30 gene to measure and correct for DNA shearing, and also to quantify input cell number. Lastly, the *env*-2LTRc assay measures unintegrated 2LTR circles by duplexing the IPDA *env* amplicon with an amplicon specific for the LTR-LTR junction; double-positive events from this assay are subtracted from the intact proviruses quantified using the IPDA. Reactions were set up in a total volume of 22 µl, with 10 µl 2X Bio-Rad ddPCR Supermix (no deoxyuridine triphosphate), primers at a final concentration of 600 nM and probes at 200 nM for the IPDA and *env*-2LTRc assays; or 500 nM primers and 250 nM probes for the RPP30 assay. Primer and probe sequences are detailed in Supplementary Table 7. Droplets were made using the Bio-Rad QX200 automated droplet generator, then thermalcycled according to the protocol detailed in Supplementary Table 8. Individual droplets were analysed for end-point fluorescence using the Bio-Rad QX200 droplet reader. Data analysis was conducted using the Quantasoft Studio software. Wells with fewer than 10,000 droplets were excluded from analysis.

### SIVmac239M barcode sequencing

Plasma viral RNA was quantified by RT–PCR before sequencing on an Illumina MiSeq sequencer as previously described^18^. For low-template samples, single genome amplification (SGA) was used, followed by direct Sanger sequencing to assess the frequency and number of unique barcodes.

### Determination of intracellular cytokine induction following SIV-Gag peptide stimulation

Cryopreserved PBMCs and lymph node mononuclear cells were thawed, rested and resuspended in RPMI 1640 medium (Corning) supplemented with 10% FBS (Peak Serum), 100 U ml^−1^ penicillin streptomycin (Corning) and 2 mM l-glutamine (Gibco) in the presence of CD107a-PEefluor660 (5 μl, clone H4A3; Invitrogen, 61-1079-42), CD49D (1 μl, clone 9F10; Invitrogen, 14-0499-82) and CD28-BUV737 (5 μl, clone CD28.2; BD Biosciences, 612815). Mononuclear cells were stimulated for 6 h at 37 °C/5% CO_2_ with SIVmac_239_ overlapping Gag peptides (NIH AIDS Reagent Program) at a concentration of 1 μg ml^−1^ in the presence of Brefeldin A (BD Biosciences, 555029) and monesin (BD Biosciences, 554724). Staphylococcal enterotoxin A & B (SEB/A-List Biologicals) stimulation at 250 ng ml^−1^ served as a positive control. Peptide diluent (1% dimethyl sulfoxide) served as the negative control. After stimulation, cells were washed and stained for cell surface antigens with the following combination of mAbs: CD3-Alexa700 (1:200, clone SP34-2; BD Biosciences, 557917), CD95-APC (1:200, clone DX2; BD Biosciences, 558814), CD4-BV711 (1:200, clone L200; BD Biosciences, 563913), CD8 PerCP-Cy5.5 (1:200, clone RPA-T8/SK1; Biolegend, 344710) and LIVE/DEAD Fixable Yellow (1 μl of 1:15 PBS dilution, Life Technologies, L34959). To detect intracellular expression of cytokines, mononuclear cells were fixed and permeabilized with a FoxP3/Transcription Factor Staining Buffer kit (Tonbo Biosciences) and stained as follows: TNF-α-BV650 (1:100, clone Mab11; Biolegend, 502938), IL-2-PE-Cy7 (1:100, clone MQ1-17H12; Biolegend, 500307), IFN-γ-PE (1:100, clone B27; BD Biosciences, 554701) and Granzyme B-BV421 (1:100, BD Biosciences, 563389). All mAbs were used at the manufacturers’ recommended test volume. Data acquisition was performed on a BD FACSymphony (BD Biosciences) driven by FACS DiVa software and analysed using FlowJo software (version 10.8; TreeStar). The frequency of SIV-specific memory CD8^+^ T cells producing single cytokines was determined after background subtraction.

### Statistical analyses

Data collection and analysis were not performed blind to the conditions of the experiments. Based on our previous data^16^ on SIV-infected ART-treated RMs, with a sample size of at least 8, we would be able to detect a significant difference between pre- and post-CD8 depletion samples in the level of plasma RNA at the 0.05 significance level with a power of 0.90. No animals or data points were excluded from the analyses. Data distribution was not formally tested. Statistical analyses, including Kruskal Wallis test, Welch’s *t*-test and Mann Whitney test were performed using GraphPad Prism v.9.0. Data presented are mean ± s.e.m., unless otherwise indicated. A *P* ≤ 0.05 was considered statistically significant.

### Mixed-effects modelling

A mixed-effects modelling approach was incorporated to fit a two-phase decay model to the combined data of CA-DNA, CA-RNA and plasma viral load. The Akaike Information Criterion (AIC) was used to compare various model structures (that is, treatment group-specific fixed effects and random effects associated with different model parameters), and the model with the lowest AIC was selected as the preferred model. See Supplementary Information for details of the mathematical model and model fitting.

### Estimating the reactivation rate

Reactivation rate was estimated using a previously developed mathematical model^18^ based on the ratio of viral growth rate to viral loads of different barcoded clonotypes. Details of the mathematical model are presented in the Supplementary Information.

### Reporting summary

Further information on research design is available in the Nature Portfolio Reporting Summary linked to this article.

### Supplementary information

Supplementary Information Supplementary Methods on the mathematical model, Figs. 1–6 and Tables 1–8.

Reporting Summary

### Source data

Source Data Excel file containing all source data for each Figure/Extended Data item.

## Figures and Tables

**Extended Data Table 1:**
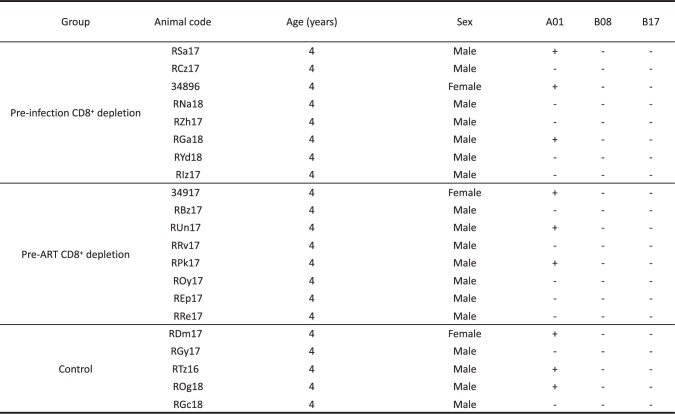
Rhesus macaques’ characteristics

## Data Availability

The raw data for all graphs generated in this study are provided in the Supplementary Information and Source Data file. Source data are provided with this paper.
