# Supplementary material for: CD8+ lymphocytes do not impact SIV reservoir establishment under ART
Source: Nat Microbiol. 2023 Jan 23;8(2):299–308. doi: 10.1038/s41564-022-01311-9 (PMC9894752; doi:10.1038/s41564-022-01311-9)
Supplement: Supplementary file 1 — Supplementary Methods on the mathematical model, Figs. 1–6 and Tables 1–8. [file 41564_2022_1311_MOESM1_ESM.pdf]

nature microbiology

Article

https://doi.org/10.1038/s41564-022-01311-9

CD8^{+} lymphocytes do not impact SIV reservoir establishment under ART

In the format provided by the authors and unedited

SUPPLEMENTARY INFORMATION

1. Mathematical model for incorporating short-lived and long-lived cells

Here we developed a mathematical model to capture the post-ART viral dynamics. In this model we included both short-lived productively infected and long-lived latently infected cells. We assumed that the total plasma virus is composed of the virus produced by short-lived productively infected cells and the virus produced by reactivated long-lived latently infected cells (Supplementary Figure 1a). Since both cell-associated viral RNA and plasma virus are produced by the infected cells, we described these two types of measurements as plasma viral load and viral RNA content per short-lived productively infected cell and long-lived latently infected cell, respectively (Supplementary Figure 1b). The mathematical model is given by

$$D(t) = \left[D_L f_S^{(\mathrm{D})} \mathrm{e}^{-\delta_{\mathrm{S}}(t-t_0)}\right] + \left[D_L \mathrm{e}^{-\delta_L(t-t_0)}\right], \tag{1}$$

$$R(t) = p_S^{(\mathrm{R})} \left[D_L f_S^{(\mathrm{D})} \mathrm{e}^{-\delta_{\mathrm{S}}(t-t_0)}\right] + p_L^{(\mathrm{R})}\left[D_L \mathrm{e}^{-\delta_L(t-t_0)}\right], \tag{2}$$

$$V(t) = p_S^{(\mathrm{V})} \left[D_L f_S^{(\mathrm{D})} \mathrm{e}^{-\delta_{\mathrm{S}}(t-t_0)}\right] + p_L^{(\mathrm{V})}\left[D_L \mathrm{e}^{-\delta_L(t-t_0)}\right], \tag{3}$$

where $D(t)$, $R(t)$, and $V(t)$ represent the total cell-associated- (CA-) DNA content, CA-RNA content, and plasma viral load, respectively. D_{L} is the DNA content associated with long-lived latently infected cells at t_0 – the day when ART is applied. $f_{\mathrm{S}}^{(\mathrm{D})}$ is the ratio of DNA content associated with short-lived infected cells to that associated with long-lived infected cells at t_0. δ_{S} and δ_{L} are the decline rates for the DNA associated with short and long-lived infected cells, respectively. $p_{\mathrm{S}}^{(\mathrm{R})}$ and $p_{\mathrm{S}}^{(\mathrm{V})}$ are CA-RNA content and plasma viral load per short-lived infected cell respectively; and $p_{\mathrm{L}}^{(\mathrm{R})}$ and $p_{\mathrm{L}}^{(\mathrm{V})}$are CA-RNA content and plasma viral load per long-lived infected cell respectively. Equations (1)-(3) provide quantitative modelling of short-lived and long-lived cell populations, so as to capture the effects of short and long-lived infected cell dynamics on total viral dynamics.

2. Model calibration

Equations (1)-(3) contain eight parameters (Supplementary Table 1). For convenience, hereafter we term the DNA content associated with short-lived infected cells as short-lived DNA, and the DNA content associated with long-lived infected cells as long-lived DNA. To estimate these parameters, we fit the mathematical model to the combined post-ART data of CA-DNA, CA-RNA, and plasma viral load during the primary infection. The model calibration was implemented under a non-linear mixed-effects modelling approach, using the *nlme* library in R (version 4.0.4). For each animal, the parameters with a random effect were taken from a normal distribution, with each parameter having its own mean (fixed effect). A diagonal random effect structure was used, where we assumed there was no correlation within the random effects. The model was fitted to the log-transformed data values, with a constant error model distributed around zero with a standard deviation σ. We compared the fits from hierarchical nonlinear mixed-effects models using the Akaike Information Criteria (AIC), and selected the optimal fit based on the lowest AIC. Using each animal as a random sample, the *nlme* function also tested whether a group effect on the parameter estimates was significant.

The best fitted mixed-effects modelling structure indicated that the following parameters were not significantly different between the three treatment groups: Long-lived DNA at ART initiation $y_{\mathrm{L}}^{(\mathrm{D})}$, the decline rate of the long-lived DNA δ_{L}, the CA-RNA content per short-lived infected cell $p_{\mathrm{S}}^{(\mathrm{R})}$, and the plasma viral load per short-lived infected cell $p_{\mathrm{S}}^{(\mathrm{V})}$. However, there was a different in the group-specific fixed effects in the following: The ratio of short to long-lived DNA at ART initiation $f_{\mathrm{S}}^{(\mathrm{D})}$ (suggesting a larger proportion of short lived DNA in CD8 depleted groups), the decline rate of the DNA associated with short-lived infected cells δ_{S} (slower decay in CD8 depleted groups), the CA-RNA content per long-lived infected cell $p_{\mathrm{L}}^{(\mathrm{R})}$ (higher in CD8 depleted), and the plasma viral load per long-lived infected cell $p_{\mathrm{L}}^{(\mathrm{V})}$(higher in CD8 depleted). Random effects were found to be significant in the estimates of $y_{\mathrm{L}}^{(\mathrm{D})}$, δ_{S}, $p_{\mathrm{S}}^{(\mathrm{V})}$, $p_{\mathrm{L}}^{(\mathrm{V})}$, and $p_{\mathrm{L}}^{(\mathrm{R})}$. The parameter estimates for the three experimental groups and for individual animals are

listed in Supplementary Table 2-5. The group-based parameter distributions and the calibrated model predictions are plotted in Supplementary Figure 2-11.

3. Mathematical model for estimating the reactivation rate

We used a previously developed mathematical approach to estimate the reactivation rate (Fennessey et al. 2017). With the assumption that all barcodes have approximately the same growth rate, the mathematical model is given by

$$RR = \frac{g(n-1)}{\sum_{i=1}^{n-1}(lnS_{i+1} - lnS_i)}, \quad (4)$$

where RR is the reactivation rate, g is the estimate of the growth rate of a single barcode (same as total viral growth rate), n is the number of barcodes used in the reactivation rate estimate, and S_i is the number of sequences for barcode i, for $i = 1,2,\dots,n$.

For each animal, the growth rate g was estimated as the maximal growth rate between any two viral load measurements. We assumed the exponential growth of virus between two neighboring measurements (Deeks et al. 2016), such that

$$V(t_{t+1}) = V(t_i)\mathrm{e}^{g_i(t_{i+1}-t_i)}, i = 1,2,\dots,m-1, \quad (5)$$

where $V(t_i)$ is the viral load at time t_i, and m is the number of measurements of viral load.

To estimate the growth rate, we first calculated the growth rate of virus between two neighboring measurements

$$g_i = \frac{lnV(t_{i+1}) - lnV(t_i)}{t_{i+1} - t_i}, i = 1,2,\dots,m-1, \quad (6)$$

where $V(t_i)$ is the viral load at time t_i. It is worth noting that for the viral load measurements, we considered the limit-of-detection data point at the latest measured time just before the rebound. The growth rate g for each animal is therefore the maximal growth rate between any two neighboring measurements from Equation (6)

$$g = \max_{i=1,2,\dots m-1} g_i. \quad (7)$$

4. Mathematical model for estimating the setpoint.

To exclude the peak of reactivation and compare the virus in a time interval that is available in most animals, we defined the setpoint as the time of infection between 30 and 75 days after the first detection of virus rebound in blood. The time-weighted $\log_{10}$ viral load at the setpoint, $\bar{V}_s$, is calculated using

$$\bar{V}_s = \frac{1}{t_e - t_s} \int_{t_s}^{t_e} \log_{10} V(t)\ \mathrm{d}t, \qquad (8)$$

where t_s and t_e are the first and last time point used for estimating the setpoint. $V(t)$ is the viral load at time t. We performed linear interpolation to the $\log_{10}$-transformed viral load data to estimate $V(t)$ between measured data points.

SUPPLEMENTARY FIGURES

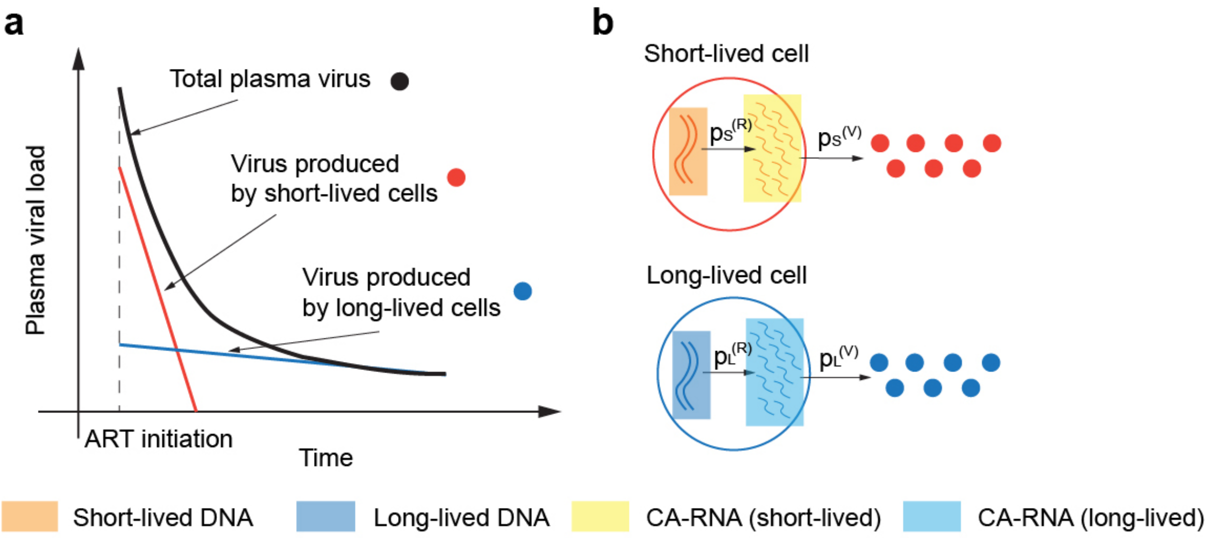

Total CA-DNA $D(t)$ = Short-lived DNA $D_S(t)$ + Long-lived DNA $D_L(t)$

Total CA-RNA $R(t)$ = RNA (short-lived) $p_S^{(R)} D_S(t)$ + RNA (long-lived) $p_L^{(R)} D_L(t)$

Total viral load $V(t)$ = Virus (short-lived) $p_S^{(V)} D_S(t)$ + Virus (long-lived) $p_L^{(V)} D_L(t)$

Supplementary Figure 1. Schematics of the mathematical model. a, Schematic of the total viral dynamics as a result of the dynamics contributed by short and long-lived infected cells, respectively. **b**, Schematic of how the short and long-lived infected cells affect the dynamics of CA-RNA and plasma virus.

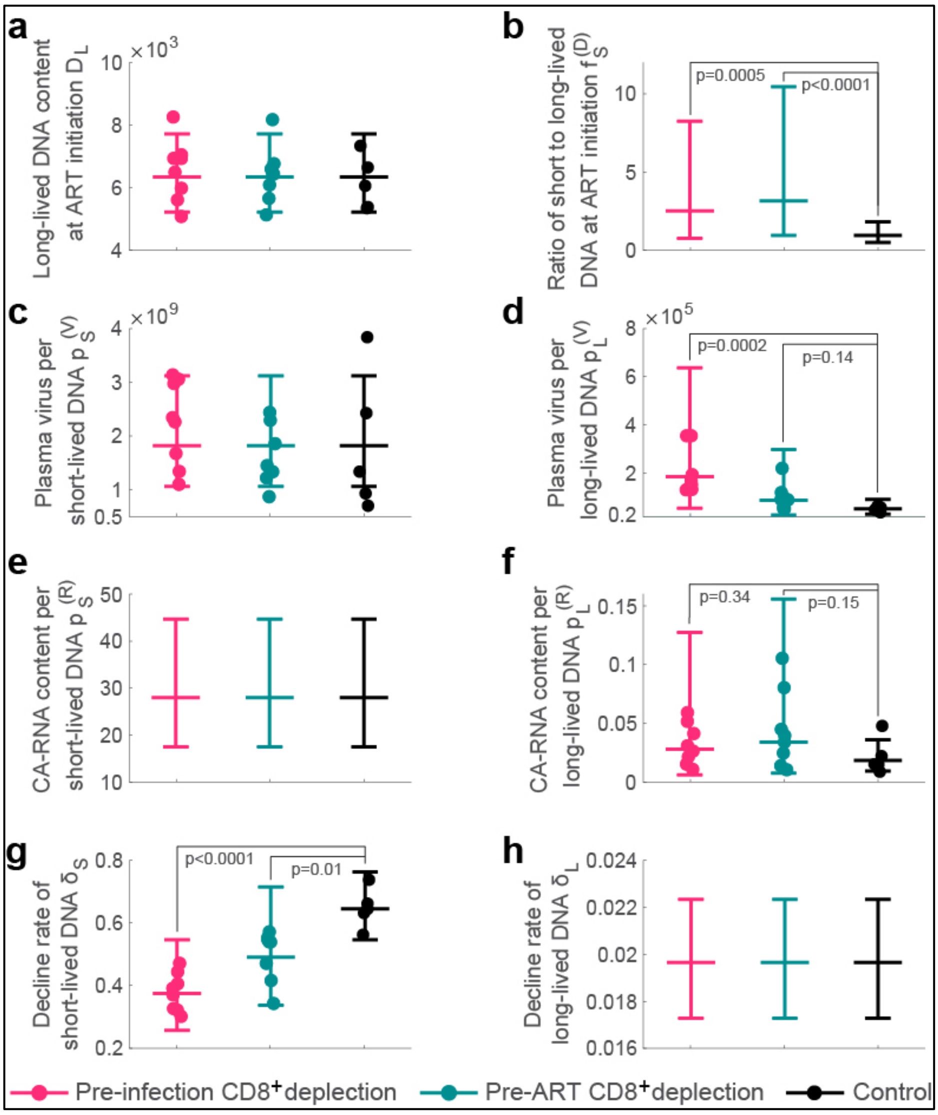

Supplementary Figure 2. Parameter estimates of the Mathematical model for short-lived and long-lived cells. Magenta color represents the Pre-Infection group (n=8 macaques), teal color represents the Pre-ART group (n=8 macaques), black color represents the control group (n=5 macaques). **a**, **c**, **e** and **h** show the same fixed effects for the three groups. **b**, **d**, **f**, and **g** show different fixed effects for different groups. For parameters with no random effect (**b**, **e**, **h**), the parameter estimates are presented as fixed effect ± 95% confidence intervals of the fixed effects. For parameters with random effects (**a**, **c**, **d**, **f**, **g**), the parameter estimates are presented as fixed effect ± 95% confidence intervals of the fixed effects, with the markers representing individual animal estimates with random effects. Statistical analysis was implicitly performed using the *nlme* library in R.

Fitting of plasma viral load

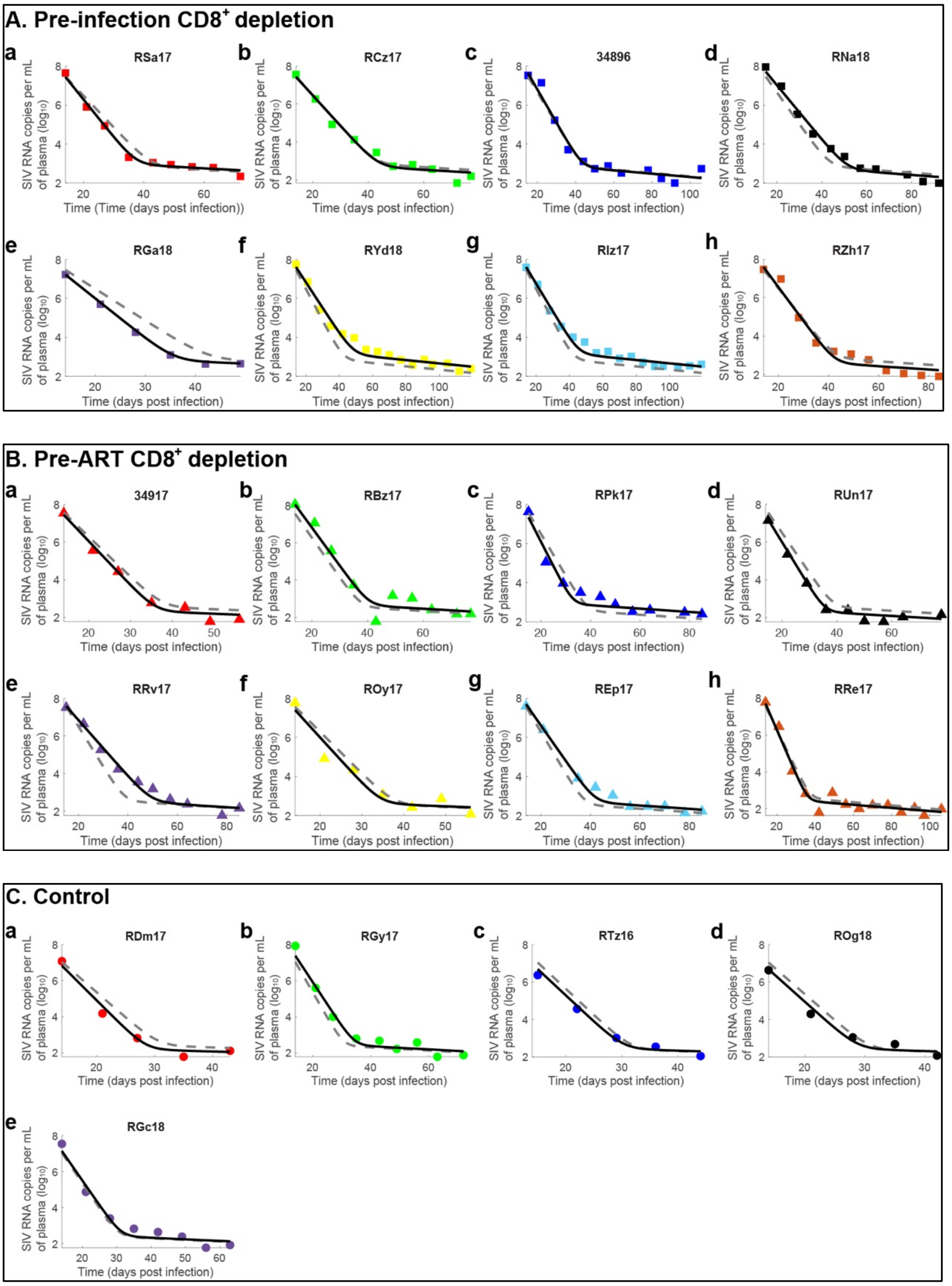

Supplementary Figure 3. Fitting results of plasma SIV viral levels with estimated parameters for the pre-infection CD8$^+$ depletion group (A), pre-ART CD8$^+$ depletion group (B), and control group (C). The black solid lines represent results with individual estimates (fixed effects + random effects). The dashed grey lines represent results with fixed effects only (group average results). The individual markers represent the CA-RNA data from PBMCs.

Fitting of cell-associated DNA

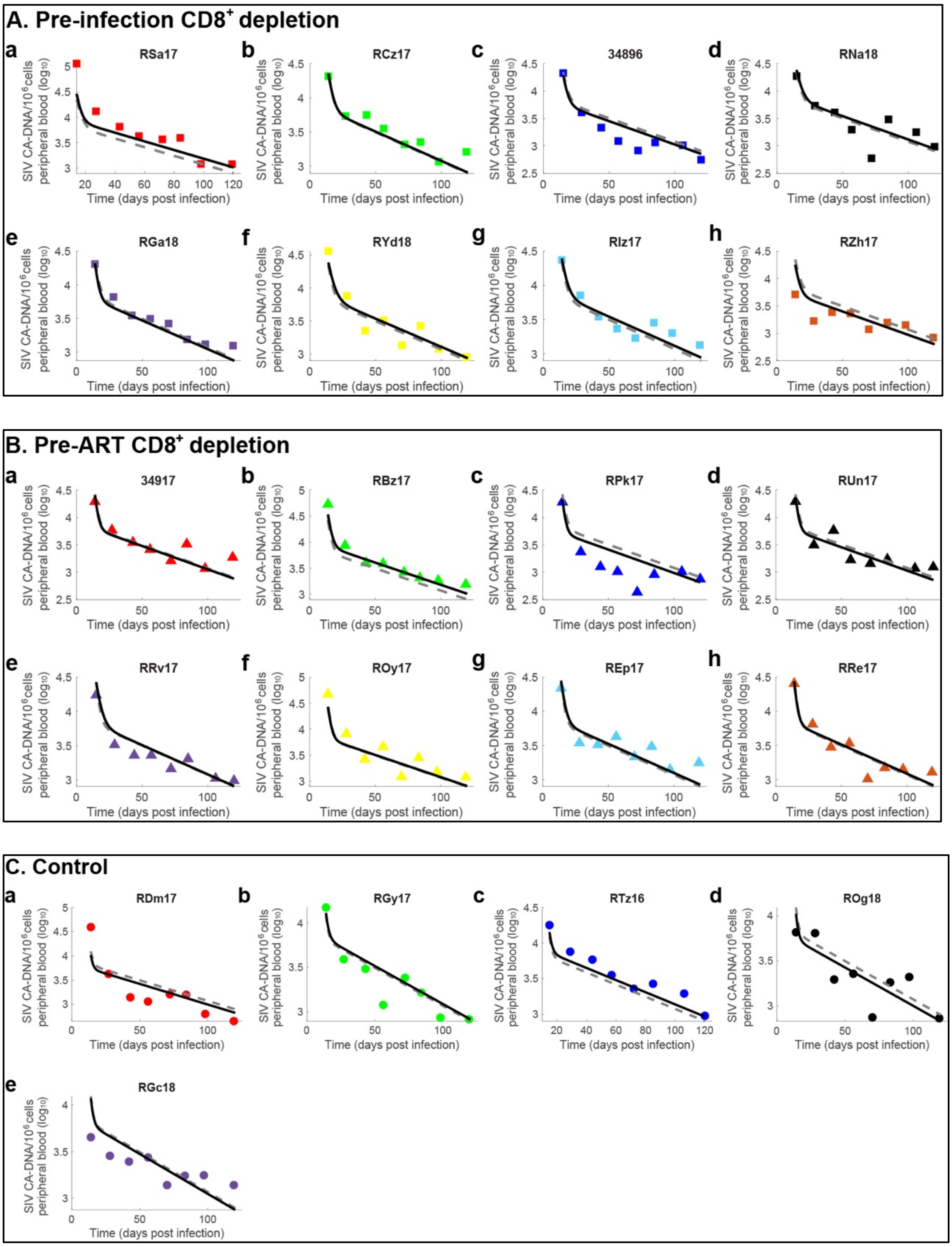

Supplementary Figure 4: Fitting results of cell-associated DNA with estimated parameters for the pre-infection CD8^{+} depletion group (A), pre-ART CD8^{+} depletion group (B), and control group (C). The black solid lines represent results with individual estimates (fixed effects + random effects). The dashed grey lines represent results with fixed effects only (group average fit). The individual markers represent the CA-DNA data from PBMCs.

Fitting of cell-associated RNA

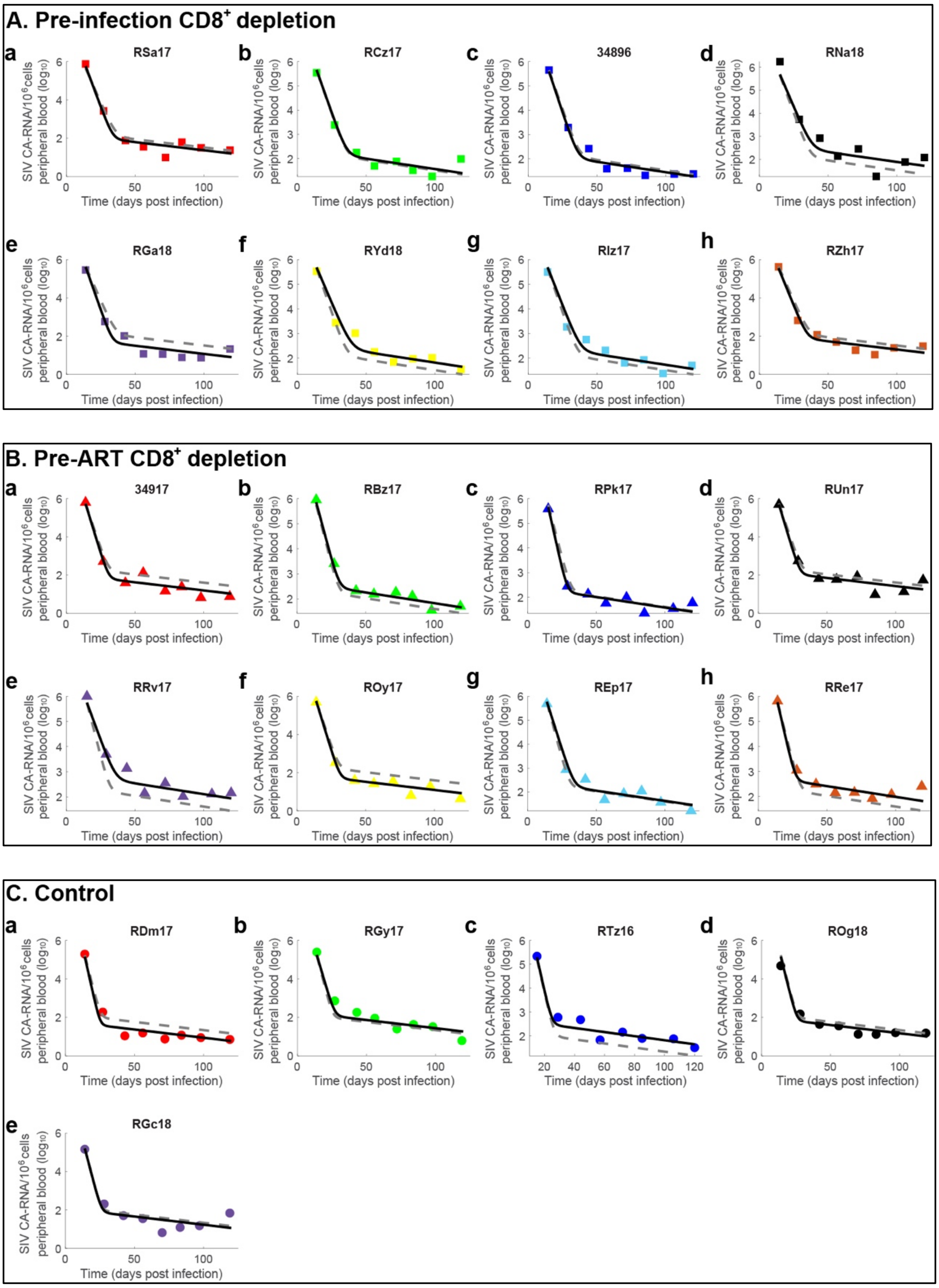

Supplementary Figure 5: Fitting results of cell-associated RNA with estimated parameters for the pre-infection CD8^{+} depletion group (A), pre-ART CD8^{+} depletion group (B), and control group (C). The black solid lines represent results with individual estimates (fixed effects + random effects). The dashed grey lines represent results with fixed effects only (group average results). The individual markers represent the CA-RNA data from PBMCs.

SUPPLEMENTARY TABLES

Parameter	Description (units)
D_{L}	DNA content associated with long-lived latently infected cells at ART initiation (CA-DNA copy/10^{6} cells)
$f_{\mathrm{S}}^{(\mathrm{D})}$	Ratio of DNA content associated with short-lived infected cells to that associated with long-lived infected cells at ART initiation (dimensionless)
δ_{S}	Decline rate of the DNA associated with short-lived infected cells (day^{-1})
δ_{L}	Decline rate of the DNA associated with long-lived infected cells (day^{-1})
$p_{\mathrm{S}}^{(\mathrm{R})}$	CA-RNA content per short-lived infected cell (CA-RNA /CA-DNA)
$p_{\mathrm{S}}^{(\mathrm{V})}$	Plasma viral load per short-lived infected cell (SIV RNA copies mL^{-1}/(CA-DNA copy/10^{6} cells))
$p_{\mathrm{L}}^{(\mathrm{R})}$	CA-RNA content per long-lived infected cell (CA-RNA /CA-DNA)
$p_{\mathrm{L}}^{(\mathrm{V})}$	Plasma viral load per long-lived infected cell (SIV RNA copies mL^{-1}/(CA-DNA copy/10^{6} cells))

Supplementary Table 1: Parameter descriptions in Equations (1)-(3).

	Pre-infection	Pre-ART	Control
Long-lived DNA at t_0 D_{L}	6.34×10^{3}		
Ratio of short to long-lived DNA at t_0 $f_{\mathrm{S}}^{(\mathrm{D})}$	2.50 (p=0.0005)	3.16 (p<0.0001)	0.95
Decline rate of short-lived DNA δ_{S}	0.38 (p<0.0001)	0.49 (p=0.01)	0.65
Decline rate of long-lived DNA δ_{L}	0.02		
CA-RNA per short-lived cell $p_{\mathrm{S}}^{(\mathrm{R})}$	27.95		
Plasma virus per short-lived cell $p_{\mathrm{S}}^{(\mathrm{V})}$	1.82×10^{9}		
CA-RNA per long-lived cell $p_{\mathrm{L}}^{(\mathrm{R})}$	0.03 (p=0.34)	0.03 (p=0.15)	0.02
Plasma virus per long-lived cell $p_{\mathrm{L}}^{(\mathrm{V})}$	1.86×10^{5} (p=0.0002)	8.67×10^{4} (0.14)	5.19×10^{4}

Supplementary Table 2: Parameter estimates for the fixed effects. The grey shaded cells indicate the same fixed effects across the three groups. The orange shaded cells indicate the estimates that are significantly different from those of the control group, with p values included in the cells. Statistical analysis was implicitly performed using the *nlme* library in R.

	RSa17	RCz17	34896	RNa18	RGa18	RYd18	RIz17	RZh17
Long-lived DNA at t_0 D_{L}	8.26×10^3	6.49×10^3	5.61×10^3	6.93×10^3	5.98×10^3	6.93×10^3	7.05×10^3	5.08×10^3
Ratio of short to long-lived DNA at t_0 $f_{\mathrm{S}}^{(\mathrm{D})}$	2.50							
Decline rate of short-lived DNA δ_{S}	0.44	0.37	0.39	0.32	0.47	0.30	0.33	0.41
Decline rate of long-lived DNA δ_{L}	0.02							
CA-RNA per short-lived cell $p_{\mathrm{S}}^{(\mathrm{R})}$	27.95							
Plasma virus per short-lived cell $p_{\mathrm{S}}^{(\mathrm{V})}$	1.34×10^9	1.67×10^9	3.06×10^9	2.98×10^9	1.10×10^9	2.34×10^9	2.26×10^9	3.14×10^9
CA-RNA per long-lived cell $p_{\mathrm{L}}^{(\mathrm{R})}$	1.52×10^{-2}	3.09×10^{-2}	2.63×10^{-2}	5.92×10^{-2}	1.07×10^{-2}	5.17×10^{-2}	4.13×10^{-2}	2.15×10^{-2}
Plasma virus per long-lived cell $p_{\mathrm{L}}^{(\mathrm{V})}$	1.68×10^5	1.32×10^5	1.93×10^5	1.31×10^5	1.52×10^5	3.55×10^5	3.55×10^5	1.30×10^5

Supplementary Table 3 Parameter estimates (fixed effects with random effects) for individual animals from the pre-infection group. The grey shaded cells indicate the same fixed effects across the three groups with no random effect. The blue shaded cell indicates the group specific fixed effect with no random effect.

	34917	RBz17	RPk17	RUn17	RRv17	ROy17	Rep17	RRe17
Long-lived DNA at t_0 D_L	6.09×10^3	8.17×10^3	5.13×10^3	5.66×10^3	6.43×10^3	6.48×10^3	6.76×10^3	6.59×10^3
Ratio of short to long-lived DNA at t_0 $f_\mathrm{S}^{(\mathrm{D})}$	3.16							
Decline rate of short-lived DNA δ_S	0.54	0.47	0.57	0.55	0.34	0.54	0.42	0.55
Decline rate of long-lived DNA δ_L	0.02							
CA-RNA per short-lived cell $p_\mathrm{S}^{(\mathrm{R})}$	27.95							
Plasma virus per short-lived cell $p_\mathrm{S}^{(\mathrm{V})}$	1.45×10^9	4.38×10^9	1.33×10^9	8.67×10^8	1.86×10^9	1.22×10^9	2.29×10^9	2.44×10^9
CA-RNA per long-lived cell $p_\mathrm{L}^{(\mathrm{R})}$	1.38×10^{-2}	4.48×10^{-2}	3.95×10^{-2}	2.47×10^{-2}	1.01×10^{-1}	1.04×10^{-2}	3.39×10^{-2}	8.04×10^{-2}
Plasma virus per long-lived cell $p_\mathrm{L}^{(\mathrm{V})}$	5.21×10^4	8.86×10^4	2.20×10^5	5.18×10^4	9.07×10^4	9.16×10^4	1.19×10^5	6.11×10^4

Supplementary Table 4 Parameter estimates (fixed effects with random effects) for individual animals from the pre-ART group. The grey shaded cells indicate the same fixed effects across the three groups with no random effect. The blue shaded cell indicates the group specific fixed effect with no random effect.

	RDm17	RGy17	RTz16	ROg18	RGc18
Long-lived DNA at t_0 D_L	5.34×10^3	6.64×10^3	7.33×10^3	5.38×10^3	6.06×10^3
Ratio of short to long-lived DNA at t_0 $f_\mathrm{S}^{(\mathrm{D})}$	0.95				
Decline rate of short-lived DNA δ_S	0.74	0.56	0.65	0.66	0.63
Decline rate of long-lived DNA δ_L	0.02				
CA-RNA per short-lived cell $p_\mathrm{S}^{(\mathrm{R})}$	27.95				
Plasma virus per short-lived cell $p_\mathrm{S}^{(\mathrm{V})}$	1.33×10^9	3.84×10^9	6.99×10^8	9.29×10^8	2.43×10^9
CA-RNA per long-lived cell $p_\mathrm{L}^{(\mathrm{R})}$	8.77×10^{-3}	2.20×10^{-2}	4.78×10^{-2}	1.48×10^{-2}	1.52×10^{-2}
Plasma virus per long-lived cell $p_\mathrm{L}^{(\mathrm{V})}$	3.69×10^4	5.74×10^4	4.78×10^4	6.36×10^4	5.85×10^4

Supplementary Table 5 Parameter estimates (fixed effects with random effects) for individual animals from the control group. The grey shaded cells indicate the same fixed effects across the three groups with no random effect. The blue shaded cell indicates the group specific fixed effect with no random effect.

SUPPLEMENTARY REFERENCES

1. Fennessey, C.M. et al. Genetically-barcoded SIV facilitates enumeration of rebound variants and estimation of reactivation rates in nonhuman primates following interruption of suppressive antiretroviral therapy. *PLoS Pathog*, **13**, e1006359 (2017).

2. Deeks, S.G. et al. International AIDS Society global scientific strategy: towards an HIV cure 2016. *Nature medicine*, **22**, 839-850 (2016).

Supplementary Figure 6. Representative flow cytometry gating strategy.

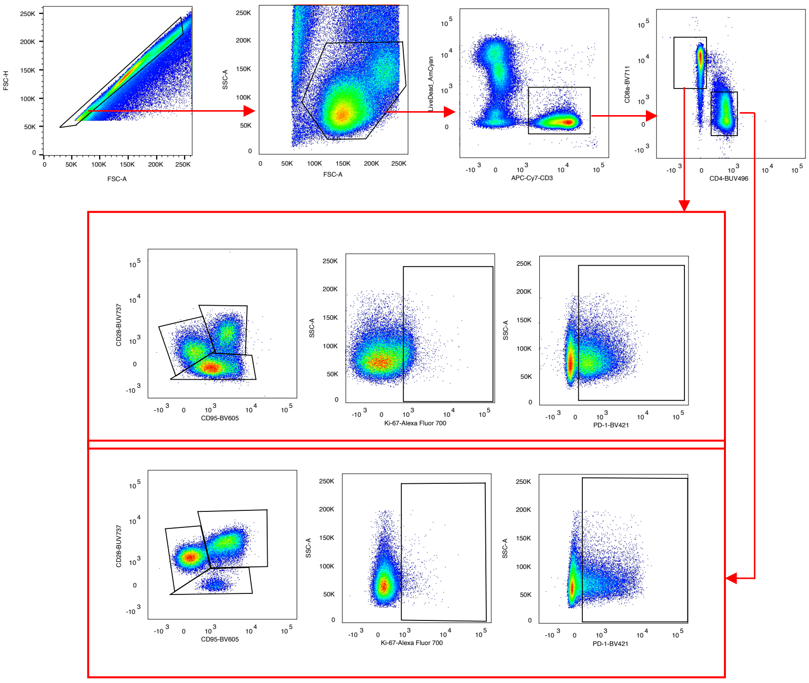

A representative flow cytometry gating strategy is shown for the immunophenotyping of fresh mononuclear cells from peripheral blood (PBMC) and lymph node (LN). Clusters of biomarkers within a single, common parental population are outlined with borders and the gating hierarchy is indicated by the directional arrows.

Supplementary Table 6. Primers and probe sequences used to quantify plasma SIV RNA, cell-associated RNA and DNA

Amplicon	Primer/Probe Name	Sequence	Fluorophore	Quencher
SHIV gag	SIVgag Fwd	5'-GCAGAGGAGGAAATTACCCAGTAC-3'	N/A	N/A
	SIVgag Rev	5'-CAATTTTACCCAGGCATTTAATGTT-3'	N/A	N/A
	SIVgag probe	5' 6FAM-TGTCCACCTGCCATTAAGCCCGA-TAMRA-3'	FAM	TAMRA
Rhesus albumin	Albumin Fwd	5'-TGCATGAGAAAACGCCAGTAA-3'	N/A	N/A
	Albumin Rev	5'-ATGGTCGCCTGTTCACCAA-3'	N/A	N/A
	Albumin Probe	5' (FAM)-AGAAAGTCACCAAATGCTGCACGGAATC-(TAMRA) 3'	FAM	TAMRA
Rhesus CD4	CD4 Fwd	5'-ACATCGTGGTGCTAGCTTTCCAGA-3'	N/A	N/A
	CD4 Rev	5'-AAGTGTAAAGGCGAGTGGGAAGGA-3'	N/A	N/A
	CD4 Probe	5' 6FAM-AGGCCTCCAGCACAGTCTATAAGAAAGAGG-TAMRA-3'	FAM	TAMRA

Supplementary Table 7. Primer and probe sequences used in the IPDA assay to quantify intact SIV genomes.

Amplicon	Primer/Probe Name	Sequence	Fluorophore	Quencher
SIVmac251 IPDA *pol*	polF	GCA GGG ATA GAG CAC ACC TTT G	N/A	N/A
	polR	CTA TGG TTT CTA CTG AAT TTG CTT GTT C	N/A	N/A
	pol intact probe	TTT CAG GTG GTG ATT C	FAM	MGBNFQ
	pol HM probe	TAG GTG GTG ATT TAT T	N/A	MGBNFQ
SIVmac239 IPDA *env*	envF	CCT CAA TAA AGC CTT GTG TAA AAT TAT C	N/A	N/A
	239 envR	GTT GTT ATT GAT TTT GTC AAT CCC	N/A	N/A
	env intact probe	TGC ATT ACT ATG AGA TGC	VIC	MGBNFQ
	env HM probe	TGC ATT ACT ATA AAA TGC	N/A	MGBNFQ
SIVmac251 2LTRc	2LTRc F	CGC CTG GTC AAC TCG GTA CTC	N/A	N/A
	2LTRc R	GGT ATG ATG CCT TCT TCC TTT TCT AAG	N/A	N/A
	2LTRc probe	CCC TGG TCT GTT AGG ACC CTT TCT GCT TTG	FAM	MGBNFQ*
Rhesus RPP30-1	RPP30-1F	AGG ATG CTC CGG GAG TAT GTA	N/A	N/A
	RPP30-1R	CCT GCT TGT CAC CTA TAT AAC AT	N/A	N/A
	RPP30-1 probe	TCA AGC TGG GAG ACG GAA GAG TCA GT	FAM	ZEN/IABkFQ
Rhesus RPP30-2	RPP30-2F	ACA GAC TCA CAC AAT TTA GG	N/A	N/A
	RPP30-2R	ACA TTC ATG CCA CTG CAC TC	N/A	N/A
	RPP30-2 probe	ACA GGG TCT CAC TTT GTT GTC CA	HEX	ZEN/IABkFQ

Supplementary Table 8. PCR conditions used in the IPDA assay to quantify intact SIV genomes.

**PCR:**	SIV IPDA		
**Step**	**Temperature**	**Time**	**# of cycles**
Enzyme activation	95°C	10 minutes	1
Denaturation	94°C	30 seconds	50
Annealing/extension	56°C	2 minutes	
Enzyme deactivation	98°C	10 minutes	1
Hold	4°C	Indefinitely	1
