# Supplementary material for: CD8+ lymphocytes do not impact SIV reservoir establishment under ART
Source: Nat Microbiol. 2023 Jan 23;8(2):299–308. doi: 10.1038/s41564-022-01311-9 (PMC9894752; doi:10.1038/s41564-022-01311-9)
Supplement: Supplementary file 2 — Reporting Summary [file 41564_2022_1311_MOESM2_ESM.pdf]

nature portfolio

Corresponding author(s): Guido Silvestri

Last updated by author(s): Nov 23, 2022

Reporting Summary

Nature Portfolio wishes to improve the reproducibility of the work that we publish. This form provides structure for consistency and transparency in reporting. For further information on Nature Portfolio policies, see our Editorial Policies and the Editorial Policy Checklist.

Statistics

For all statistical analyses, confirm that the following items are present in the figure legend, table legend, main text, or Methods section.

n/a	Confirmed	
☐	☒	The exact sample size (*n*) for each experimental group/condition, given as a discrete number and unit of measurement
☐	☒	A statement on whether measurements were taken from distinct samples or whether the same sample was measured repeatedly
☐	☒	The statistical test(s) used AND whether they are one- or two-sided<br>*Only common tests should be described solely by name; describe more complex techniques in the Methods section.*
☐	☒	A description of all covariates tested
☐	☒	A description of any assumptions or corrections, such as tests of normality and adjustment for multiple comparisons
☐	☒	A full description of the statistical parameters including central tendency (e.g. means) or other basic estimates (e.g. regression coefficient) AND variation (e.g. standard deviation) or associated estimates of uncertainty (e.g. confidence intervals)
☐	☒	For null hypothesis testing, the test statistic (e.g. *F*, *t*, *r*) with confidence intervals, effect sizes, degrees of freedom and *P* value noted<br>*Give P values as exact values whenever suitable.*
☒	☐	For Bayesian analysis, information on the choice of priors and Markov chain Monte Carlo settings
☒	☐	For hierarchical and complex designs, identification of the appropriate level for tests and full reporting of outcomes
☒	☐	Estimates of effect sizes (e.g. Cohen's *d*, Pearson's *r*), indicating how they were calculated

Our web collection on statistics for biologists contains articles on many of the points above.

Software and code

Policy information about availability of computer code

Data collection	Flow cytometry data were collected using FACS Diva V8.01 on a LSR II (BD Biosciences). Barcode RNA sequencing was performed on a an Illumina MiSeq sequencer. Plasma viremia and cell-associated SIV-DNA and RNA were collected using the Applied Biosystem 7500 Real Time PCR.
Data analysis	FlowJo V10.8 (Tree Star); Graph Pad Prism V9.0; R V4.1.2

For manuscripts utilizing custom algorithms or software that are central to the research but not yet described in published literature, software must be made available to editors and reviewers. We strongly encourage code deposition in a community repository (e.g. GitHub). See the Nature Portfolio guidelines for submitting code & software for further information.

Data

Policy information about availability of data

All manuscripts must include a data availability statement. This statement should provide the following information, where applicable:

- Accession codes, unique identifiers, or web links for publicly available datasets
- A description of any restrictions on data availability
- For clinical datasets or third party data, please ensure that the statement adheres to our policy

The data that support the findings of this study are provided in the Supplementary Information/Source Data file.

Human research participants

Policy information about studies involving human research participants and Sex and Gender in Research.

Reporting on sex and gender	Not applicable
Population characteristics	Not applicable
Recruitment	Not applicable
Ethics oversight	Not applicable

Note that full information on the approval of the study protocol must also be provided in the manuscript.

Field-specific reporting

Please select the one below that is the best fit for your research. If you are not sure, read the appropriate sections before making your selection.

☒ Life sciences ☐ Behavioural & social sciences ☐ Ecological, evolutionary & environmental sciences

For a reference copy of the document with all sections, see nature.com/documents/nr-reporting-summary-flat.pdf

Life sciences study design

All studies must disclose on these points even when the disclosure is negative.

Sample size	Based on our previous data on SIV-infected ART-treated Rhesus macaques, with a sample size of at least 8, we would be able to detect a significant difference between pre- and post-CD8 depletion samples in the level of plasma RNA at the 0.05 significance level with a power of 0.90.
Data exclusions	No data exclusion was applied to this study.
Replication	The use of nonhuman primates precludes the ability to replicate experiments. Sample size were chosen to maximize the likelihood of detecting statistical differences.
Randomization	Age, weight, sex, A01 status were all controlled for when allocate animals into experimentals groups.
Blinding	Blinding of the investigators was not possible as they were responsible for the distribution of compounds for administration and treatment stratification.

Reporting for specific materials, systems and methods

We require information from authors about some types of materials, experimental systems and methods used in many studies. Here, indicate whether each material, system or method listed is relevant to your study. If you are not sure if a list item applies to your research, read the appropriate section before selecting a response.

Materials & experimental systems

n/a	Involved in the study	
☐	☒	Antibodies
☒	☐	Eukaryotic cell lines
☒	☐	Palaeontology and archaeology
☐	☒	Animals and other organisms
☒	☐	Clinical data
☒	☐	Dual use research of concern

Methods

n/a	Involved in the study	
☒	☐	ChIP-seq
☐	☒	Flow cytometry
☒	☐	MRI-based neuroimaging

Antibodies

Antibodies used	All antibodies were used as per manufacturer's recommendation. CCR5-BV650 (5uL, clone 3A9; BD Biosciences 564999) and CCR7 FITC (5uL, clone 150503; BD Biosciences, 561271), LIVE/DEAD aqua viability dye (1 µL of 1:20 PBS dilution, ThermoFisher, L34966), CD3-APC-Cy7 (5uL, clone SP34-2; BD Biosciences 557757), CD4-BUV496 (5uL, clone SK3, BD Biosciences 564651), CD8α-BV711 (5uL, clone RPA-T8; Biolegend 301044), CD8β-PE-Cy7 (5uL, clone SIDI8BEE; Invitrogen 14-5273-82), CD45RA-Pe-Cy5 (5uL, clone 5H9; BD

Biosciences 552888), CD62L-BV786 (5uL, clone SK11; BD Biosciences 565311), CD95-BV605 (5uL, clone DX2; Biolegend 305628), PD-1-BV421 (5uL, clone EH12.2H7; Biolegend 329920), CD14-BV510 (5uL, clone M5E2; Biolegend 301842), CD20-BV510 (5uL, clone 2H7; Biolegend 302340), NKG2A (also known as CD159a)-APC (5uL, clone Z199; Beckman Coulter, A60797), CD28-BUV737 (5uL, clone CD28.2; BD Biosciences 612815), CD69-Pe-CF594 (5uL, clone FN50; BD Biosciences 562617), CD25-BUV395 (5uL, clone 2A3; BD Biosciences 564034), HLA-DR-PerCP-Cy5.5 (5uL, clone G46-6; BD Biosciences 552764), Ki-67-AF700 (5uL, clone B56; BD Biosciences, 561277), CD107a-PEefluor660 (5uL, clone H4A3; Invitrogen 61-1079-42), CD49D (1uL, clone 9F10; Invitrogen 14-0499-82), CD3-Alexa700 (5uL, clone SP34-2; BD Biosciences 557917), CD95-APC (5uL, clone DX2; BD Biosciences 558814), CD4-BV711 (5uL, clone L200; BD Biosciences 563913), CD8 PerCP-Cy5.5 (5uL, clone RPA-T8/SK1; Biolegend 344710), LIVE/DEADTM Fixable Yellow (1 µL of 1:15 PBS dilution, Life Technologies Life Technologies L34959), TNF-[illegible]-BV650 (5uL, clone Mab11; Biolegend 502938), IL-2-PE-Cy7 (5uL, clone MQ1-17H12; Biolegend 500307), IFN-γ-PE (5uL, clone B27; BD Biosciences 554701), Granzyme B-BV421 (5uL, BD Biosciences 563389).

Validation: Antibodies were validated in previous Nonhuman primate studies (see Reference #17, 44-46) and the NIH Nonhuman Primate Reagent Resource.

Animals and other research organisms

Policy information about studies involving animals; ARRIVE guidelines recommended for reporting animal research, and Sex and Gender in Research

Laboratory animals	21 Indian Rhesus macaques (RM; Macaca mulatta), housed at Emory National Primate Research Center (3 females, 18 males; 3-4 years at the start of the study.
Wild animals	The study did not involve wild animals
Reporting on sex	N/A
Field-collected samples	No field-collected samples were used in the study
Ethics oversight	All the procedures were approved by the Emory University Institutional Animal Care and Use Committee (IACUC). Animal care facilities at Emory National Primate Research Center are accredited by the U.S. Department of Agriculture (USDA) and the Association for Assessment and Accreditation of Laboratory Animal Care (AAALAC) International.

Note that full information on the approval of the study protocol must also be provided in the manuscript.

Flow Cytometry

Plots

Confirm that:

☒ The axis labels state the marker and fluorochrome used (e.g. CD4-FITC).

☒ The axis scales are clearly visible. Include numbers along axes only for bottom left plot of group (a 'group' is an analysis of identical markers).

☒ All plots are contour plots with outliers or pseudocolor plots.

☒ A numerical value for number of cells or percentage (with statistics) is provided.

Methodology

Sample preparation	Blood and lymph node (LN) biopsies collection were performed longitudinally and at necropsy. Blood samples were used for a complete blood count and routine chemical analysis, and plasma was separated by centrifugation within 1 hour of phlebotomy. Peripheral blood mononuclear cells (PBMCs) were isolated from whole blood by density gradient centrifugation. For LN biopsies, the skin over the axillary or inguinal region was clipped and surgically prepped. An incision was then made in the skin over the LN, which was exposed by blunt dissection and excised over clamps. LNs were then homogenized and passed through a 70-um cell strainer to isolate lymphocytes. All samples were processed, fixed (1% paraformaldehyde), and analyzed within 24 hours of collection.
Instrument	LSR II (BD Biosciences)
Software	FACS Diva V8.01. The data were further analyzed using FlowJo V10.8 (TreeStar).
Cell population abundance	FACS sorting of cellular subsets was not performed.
Gating strategy	Mononuclear cells were defined as laying on the diagonal of FSC-A versus FSC-H, and lymphocytes were gated fron FSC-A versus SSC-A. CD4+ and CD8+ T cells were pre-gated as live CD3+ lymphocytes, and memory subsets were gated as CD95 +CD28+/-.

☒ Tick this box to confirm that a figure exemplifying the gating strategy is provided in the Supplementary Information.
